# Immune checkpoint inhibitor therapy for gastric cancer: current status, therapeutic challenges, and future prospects

**DOI:** 10.3389/fimmu.2026.1716934

**Published:** 2026-02-26

**Authors:** Penghui Liu, Na Li, Jiwu Guo, Gengyu Tong Zhao, Jizhen Wang, Ziyuan Mou, Jie Mao

**Affiliations:** 1 Lanzhou University Second Hospital, The General Surgery Department, Lanzhou, China; 2 Northeastern University, Boston, MA, United States

**Keywords:** efficacy and safety, gastric cancer, immune checkpoint inhibitors, immunotherapy, personalized treatment

## Abstract

Gastric cancer is among the most prevalent malignant tumors of the digestive system worldwide. In recent years, immune checkpoint inhibitors (ICIs) have achieved substantial advances in the treatment of gastric cancer. By blocking the PD-1/PD-L1 and CTLA-4 signaling pathways, ICIs enhance antitumor immune responses and offer novel therapeutic options for patients. However, their clinical application continues to face significant challenges, including therapeutic resistance, immune-related adverse events, the lack of reliable biomarkers, and an immunosuppressive tumor microenvironment. This narrative review summarizes recent advances in ICIs-based therapies for gastric cancer, provides an in-depth analysis of existing clinical challenges, and highlights key future research directions, including biomarker discovery, development of predictive models, optimization of combination regimens, targeting of resistance mechanisms, modulation of the tumor-associated microbiota, and improved toxicity management. Moving forward, efforts should focus on advancing immunotherapy toward individualized and precision-based approaches to maximize both efficacy and safety, thereby enabling further optimization and breakthroughs in gastric cancer immunotherapy.

## 1 Introduction

Gastric cancer is among the most common malignant tumors of the digestive system worldwide, ranking fifth in both incidence and mortality, with particularly high disease burden observed in regions such as East Asia (1, 2). Because early clinical symptoms are often subtle and nonspecific, most patients are diagnosed at an advanced stage—either locally progressed or metastatic—resulting in an overall poor prognosis (3). Current treatment strategies for gastric cancer include surgery, chemotherapy, radiotherapy, and targeted therapy; however, therapeutic efficacy remains limited in patients with advanced or recurrent metastatic disease, underscoring the urgent need for novel strategies to improve survival outcomes (4).

In recent years, advances in tumor immunology have highlighted immune checkpoint inhibitors (ICIs) as a promising new immunotherapeutic approach for gastric cancer (5, 6). ICIs exert their effects primarily by blocking the programmed death-1 (PD-1), programmed death-ligand 1 (PD-L1), and cytotoxic T-lymphocyte–associated antigen 4 (CTLA-4) signaling pathways, thereby relieving immune suppression and enhancing antitumor immune responses (7, 8). Clinical studies have demonstrated that ICIs can significantly prolong survival in patients with advanced gastric cancer, with particularly notable benefits observed in those with high PD-L1 expression or microsatellite instability–high (MSI-H) tumors (9). However, compared with “immunologically hot” tumors such as lung cancer or melanoma, gastric cancer typically exhibits the characteristics of an “immunologically cold” tumor, including insufficient T-cell infiltration, low tumor antigenicity, and a markedly immunosuppressive microenvironment (10–12). Consequently, the objective response rate (ORR) to ICI monotherapy remains low, and clinical benefits are restricted to a relatively small subset of patients (13). Moreover, resistance mechanisms, immune-related adverse events (irAEs), the lack of reliable biomarkers, and continued immunosuppression within the tumor microenvironment (TME) further limit the clinical application (14, 15).

To overcome these limitations, improve response rates, and broaden the eligible patient population, recent strategies have focused on combining ICIs with chemotherapy, radiotherapy, targeted agents, anti-angiogenic drugs, and novel immune modulators (16). At the same time, the rise of precision medicine has driven ongoing efforts to identify and validate novel predictive biomarkers (17). In this narrative review, we summarize recent advances in the application of ICIs for gastric cancer, with a particular focus on key mechanisms limiting therapeutic efficacy, the clinical potential of combination strategies, and emerging directions in biomarker research. The aim is to provide a theoretical foundation for optimizing immunotherapeutic approaches and facilitating their clinical translation in gastric cancer.

## 2 Mechanism of action of ICIs

**Figure 1:**
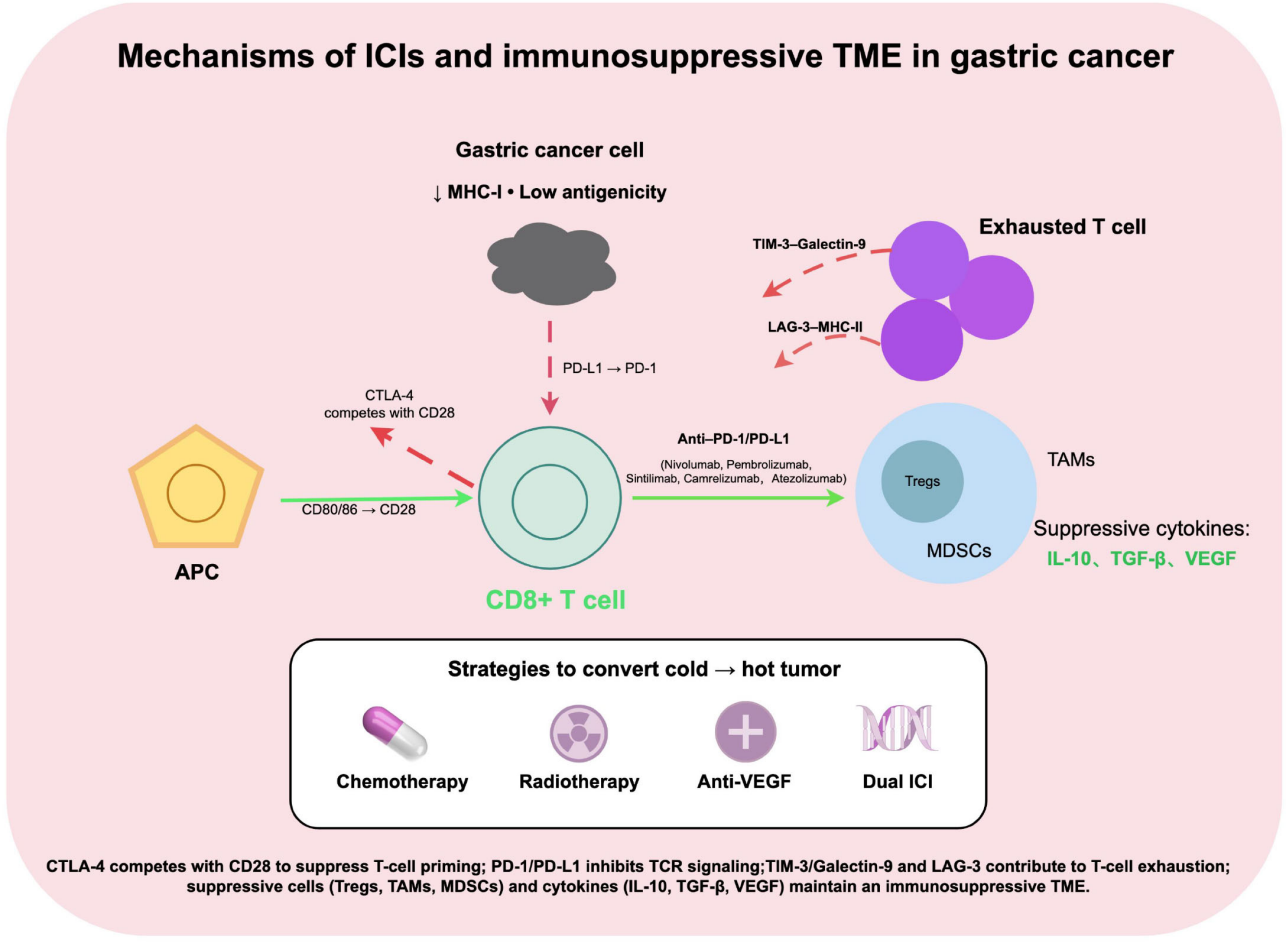
Mechanisms of ICIs and immunosuppressive TME in gastric cancer. Reduced MHC-I expression and immune checkpoint signaling (CTLA-4, PD-1/PD-L1, TIM-3, LAG-3) limit T-cell activation and induce exhaustion. The TME is shaped by suppressive cells (Tregs, immunosuppressive TAMs, MDSCs) and cytokines (IL-10, TGF-β, VEGF). Combination strategies aim to convert immunologically **“**cold**”** tumors into **“**hot**”** immune-responsive tumors.

ICIs enhance T cell–mediated antitumor immune responses by relieving immune suppression, modulating the TME, and activating the immune system, thereby strengthening the body’s ability to recognize and eliminate tumor cells (18, 19). Among the various immune checkpoint pathways, PD-1/PD-L1 and CTLA-4 are the most extensively studied and clinically applied in gastric cancer immunotherapy (**Figure 1**).

PD-1 is an immune checkpoint receptor expressed on the surface of T cells, and its primary ligand, PD-L1, is highly expressed on gastric cancer cells as well as antigen-presenting cells. The interaction between PD-1 and PD-L1 induces T-cell dysfunction, diminishes T-cell activation, promotes apoptosis, and reduces cytokine secretion, thereby attenuating the antitumor immune response (20–22). Moreover, this signaling pathway enhances the immunosuppressive function of regulatory T cells (Tregs), further contributing to the formation of an immunosuppressive TME (23). PD-1/PD-L1 inhibitors restore CD8^+^ T-cell activity and enhance their capacity to recognize and eliminate tumor cells through blockade of this pathway (24). Currently, commonly used PD-1 inhibitors in gastric cancer treatment include nivolumab (25), pembrolizumab (26), sintilimab (27), and camrelizumab (28), while the PD-L1 inhibitor atezolizumab (29) is also widely utilized.

CTLA-4 is another immune checkpoint receptor expressed on activated T cells and Tregs, primarily exerting a negative regulatory role during the early stages of T-cell activation (30, 31). CTLA-4 functions by competitively binding to the co-stimulatory molecules CD80/CD86, thereby inhibiting the B7-1/B7-2–CD28 co-stimulatory signaling pathway, reducing T-cell proliferation, and limiting the antitumor immune response (32, 33). Additionally, CTLA-4 activation can enhance Treg function, further suppressing the antitumor activity of effector T cells (34). CTLA-4 inhibitors, such as ipilimumab, can relieve T-cell suppression and restore antitumor activity by blocking this pathway, and they have demonstrated clinical efficacy in various solid tumors (35, 36).

Beyond PD-1/PD-L1 and CTLA-4, several additional immune checkpoint molecules are present in the gastric cancer immune microenvironment, including T-cell immunoglobulin and mucin-domain–containing-3 (TIM-3) and lymphocyte activation gene 3 (LAG-3). TIM-3 induces T-cell exhaustion and promotes immune cell apoptosis by binding to ligands such as Galectin-9, thereby suppressing antitumor immune responses (37, 38). LAG-3 inhibits T-cell proliferation and effector functions by interacting with MHC class II molecules, thereby reinforcing the immunosuppressive TME (39). Several clinical trials are currently investigating novel ICIs targeting these molecules. Dual immune checkpoint blockade strategies, such as PD-1 plus LAG-3 or PD-1 plus TIM-3 inhibition, have shown potential to enhance antitumor efficacy (40, 41).

Overall, ICIs alleviate T-cell immune suppression and activate antitumor responses through multiple molecular targets and signaling pathways, forming the fundamental mechanistic basis of current gastric cancer immunotherapy. With the ongoing discovery of novel targets and the development of combination strategies, the therapeutic potential of ICIs in gastric cancer continues to expand.

## 3 Advances in the application of ICIs in gastric cancer treatment

ICIs represented by PD-1 and PD-L1 inhibitors have achieved significant progress in the treatment of gastric cancer. With the advancement of clinical trials, the application of ICIs has expanded from advanced metastatic gastric cancer to the perioperative setting. They have demonstrated tremendous potential in combination with chemotherapy, radiotherapy, targeted therapy, and multi-ICIs regimens, becoming a crucial component of comprehensive gastric cancer treatment (25, 42) (**Table 1**).

**Table 1:** Landmark clinical trials of ICIs in gastric cancer.

Study	Phase	ICI agent	Combination regimen	Patient population	Key outcomes
KEYNOTE-059	Second-line or later monotherapy	Pembrolizumab	Monotherapy	PD-L1– positive	ORR 15.5%
ATTRACTION-2	Third-line or later monotherapy	Nivolumab	Monotherapy	Unselected	OS 5.3 vs 4.1 month
KEYNOTE-061	Second-line monotherapy	Pembrolizumab	vs. Paclitaxel	CPS≥10	CPS≥10: OS 10.4 vs 8.0 month
CheckMate-649	First-line + chemotherapy	Nivolumab	XELOX/FOLFOX	CPS≥5	OS 14.4 vs 11.1 month PFS 7.7 vs 6.0 month
ORIENT-16	First-line + chemotherapy	Sintilimab	CAPOX	CPS≥5	OS 15.3 vs 12.5 month
ATTRACTION-4	First-line + chemotherapy	Nivolumab	XELOX	Japanese population	PFS 10.5 vs 8.3 month
JAVELIN Gastric 300	Third-line monotherapy	Avelumab	vs. chemotherapy	Post-multiline failure	No significant difference in OS, PFS, or ORR
CheckMate-577	Adjuvant therapy	Nivolumab	Post-CRT + surgery	Resected GEJ adenocarcinoma after neoadjuvant CRT	DFS 22.4 vs 11.0 month
KEYNOTE-585	Neoadjuvant therapy	Pembrolizumab	+ chemotherapy	Resectable locally advanced	Ongoing
MATTERHORN	Neoadjuvant therapy	Durvalumab	+ FLOT	Resectable locally advanced	Ongoing
KEYNOTE-811	First-line HER2-positive	Pembrolizumab	Trastuzumab + chemotherapy	HER2^+^/PD-L1^+^	ORR 74.4% vs 51.9%
LEAP-005	Targeted + immunotherapy	Pembrolizumab	+ Lenvatinib	Multi-tumor cohort (incl. GC)	ORR 10%, DCR >60%
CheckMate-032	Dual ICIs combination	Nivolumab + Ipilimumab	Dual checkpoint blockade	Advanced gastric cancer	Increased ORR; irAEs up to 26%
Neo-PLANET II	Neoadjuvant CRT + immunotherapy	Camrelizumab	Synchronous CRT	Locally advanced gastric adenocarcinoma	Improved pCR and R0 resection rates; manageable safety
RELATIVITY-047	Novel checkpoint combination	Nivolumab + Relatlimab	PD-1 + LAG-3 blockade	Early phase (melanoma)	gastric cancer exploration ongoing

### 3.1 Advanced or metastatic gastric cancer: breakthroughs in first-line treatment

In advanced or metastatic gastric cancer, ICIs, either as monotherapy or in combination with other treatments, have become an important therapeutic option. The KEYNOTE-059 trial was the first to demonstrate the efficacy of pembrolizumab in previously treated patients with PD-L1–positive gastric cancer, reporting an ORR of 15.5%. Some patients achieved durable responses, which encouraged further exploration of ICIs in gastric cancer (43). However, the subsequent ATTRACTION-2 study showed that nivolumab monotherapy achieved an ORR of only 11% in advanced gastric cancer, indicating that most patients are not sensitive to ICI monotherapy (44).Similarly, the KEYNOTE-061 trial demonstrated that pembrolizumab did not provide a significant survival advantage compared with chemotherapy, further limiting the clinical application of ICI monotherapy (45). Based on these findings, clinical research began to explore combination strategies involving ICIs and chemotherapy. The CheckMate-649 trial enrolled patients with previously untreated advanced gastric or gastroesophageal junction adenocarcinoma and demonstrated that, in those with high PD-L1 expression (CPS ≥ 5), nivolumab combined with XELOX or FOLFOX significantly prolonged median overall survival (OS) (14.4 vs. 11.1 months, HR = 0.71) and progression-free survival (PFS) (7.7 vs. 6.0 months, HR = 0.68), as well as improved ORR (60% vs. 45%), establishing nivolumab plus chemotherapy as a new first-line standard for advanced gastric cancer (25, 46). The ORIENT-16 study further validated the efficacy of sintilimab combined with CAPOX in a Chinese population, showing a significant improvement in OS among patients with PD-L1 CPS ≥ 5 (15.3 vs. 12.5 months, HR = 0.70) (47). The ATTRACTION-4 trial conducted in a Japanese population also supported the clinical value of nivolumab plus chemotherapy, demonstrating a significant extension of PFS (10.5 vs. 8.3 months, HR = 0.68) (48).Currently, ICIs-based combination chemotherapy has been incorporated into the first-line treatment recommendations for patients with high PD-L1 expression in guidelines from NCCN, ESMO, CSCO, and other major oncology societies.

#### 3.1.1 Clinical implications

- Who benefits/in what context: In previously untreated advanced or metastatic gastric/GEJ adenocarcinoma, the clearest first-line benefit from adding PD-1 blockade is seen in patients with higher PD-L1 expression (e.g., CPS ≥5), supporting PD-1 inhibitor + platinum/fluoropyrimidine chemotherapy as a preferred option in this subgroup.
- Practical interpretation: Given the limited activity of ICI monotherapy in most patients, first-line practice is primarily driven by combination-based strategies rather than monotherapy.
- What remains uncertain: Uncertainties include the optimal PD-L1 cutoff across assays, the benefit in lower-CPS subgroups, and the efficacy–toxicity tradeoff. Regional differences (OS vs PFS signals) may also reflect post-progression therapy and population/chemo-backbone heterogeneity.

### 3.2 Second and later-line therapy: patient selection as the key determinant

In the second-line (after progression on first-line therapy) and later-line settings (third-line or beyond, typically after failure of ≥2 prior systemic regimens), ICI monotherapy remains an important area of investigation, but overall outcomes are characterized by low response rates and a limited population deriving clinical benefit. The KEYNOTE-061 study compared the efficacy of pembrolizumab with paclitaxel in the second-line setting and demonstrated that pembrolizumab significantly prolonged OS only in patients with PD-L1 CPS ≥ 10 (median OS: 10.4 vs. 8.0 months, HR = 0.82), while no significant difference was observed in the overall population, suggesting that high PD-L1 expression may be a critical determinant of immunotherapy benefit (45). The ATTRACTION-2 trial enrolled patients with previously treated advanced gastric cancer in an East Asian population and showed that nivolumab significantly prolonged OS (median OS: 5.3 vs. 4.1 months, HR = 0.63). Although the ORR was relatively low (11%), a subset of patients achieved long-term survival, with a 2-year survival rate of 10.6%. This study was the first phase III trial to demonstrate a survival benefit of ICIs in the later-line treatment of gastric cancer without prior PD-L1 selection, leading to the approval of nivolumab as a third-line treatment option in Asia (44–49). However, the JAVELIN Gastric 300 trial further underscored the limitations of ICI monotherapy. The study evaluated the efficacy of avelumab (an anti–PD-L1 antibody) as third-line therapy but failed to demonstrate a survival advantage over chemotherapy, with no statistically significant differences in OS, PFS, or ORR. These findings reinforce the notion that the clinical benefit of immunotherapy is limited in unselected patient populations (50). Therefore, the efficacy of ICI monotherapy in the second- and later-line treatment of gastric cancer is markedly influenced by individual immune characteristics, particularly PD-L1 expression, MSI-H, and tumor mutational burden (TMB-H). Clinical evidence suggests that these biomarkers may predict higher immunogenicity and improved treatment responses, and they have gradually become the foundation of precision patient selection.

#### 3.2.1 Clinical implications

- Who benefits/in what context: In the second- and later-line setting, benefit from ICI monotherapy is most consistently enriched in biomarker-selected patients—particularly PD-L1 CPS ≥10 (KEYNOTE-061), and potentially MSI-H/TMB-H subsets—supporting biomarker-informed patient selection.
- Practical interpretation: Later-line PD-1 monotherapy can yield modest ORR but durable benefit in a minority, as illustrated by ATTRACTION-2 (including long-term survivors), which underpins its later-line use in some regions (e.g., Asia).
- What remains uncertain: Results are inconsistent in unselected populations, and optimal biomarker cutoffs/assays remain unsettled. Regional approvals and post-progression treatment patterns may further influence survival signals.

### 3.3 Perioperative therapy: exploring neoadjuvant and adjuvant strategies

ICIs have also shown promising potential in the perioperative treatment of locally advanced gastric cancer. In the adjuvant setting, the CheckMate-577 trial enrolled patients with locally advanced esophageal or gastroesophageal junction adenocarcinoma who had received neoadjuvant chemoradiotherapy followed by curative resection. The results demonstrated that nivolumab as postoperative adjuvant therapy significantly prolonged disease-free survival (DFS) (22.4 vs. 11.0 months, HR = 0.69), providing strong clinical evidence supporting the use of adjuvant immunotherapy after gastric cancer surgery (51). In the neoadjuvant setting, two pivotal phase III trials—KEYNOTE-585 and MATTERHORN—are currently evaluating the safety and efficacy of pembrolizumab combined with chemotherapy and durvalumab combined with the FLOT regimen, respectively, in patients with resectable gastric or gastroesophageal junction cancer (52, 53). Furthermore, several studies have shown that combining ICIs with neoadjuvant chemotherapy can enhance tumor immunogenicity and induce significant tumor regression, enabling patients with initially borderline-resectable or unresectable disease to become eligible for surgery. In some cases, this approach can even achieve a pathological complete response (pCR), thereby expanding therapeutic options for patients with advanced disease (54, 55). Research on ICIs in the perioperative setting is currently advancing rapidly. It is expected to achieve further breakthroughs in immunotherapy for gastric cancer in the future.

#### 3.3.1 Clinical implications

- Who benefits/in what context: The most robust perioperative evidence to date comes from CheckMate-577, where adjuvant nivolumab after neoadjuvant chemoradiotherapy and curative resection improved DFS in esophageal/GEJ adenocarcinoma; therefore, extrapolation to pure gastric cancer should be interpreted cautiously.
- Practical interpretation: For resectable gastric/GEJ cancer, perioperative immunotherapy remains practice-evolving, with pivotal phase III trials (e.g., KEYNOTE-585, MATTERHORN) testing ICI plus chemotherapy/FLOT; routine adoption should await mature phase III outcomes and guideline updates.
- What remains uncertain: Key gaps include the optimal timing (neoadjuvant vs adjuvant vs perioperative), biomarker-based selection, and the balance between incremental efficacy and perioperative feasibility/toxicity, despite early signals of enhanced regression/pCR in some studies.

### 3.4 HER2-positive population: synergistic effects of immunotherapy and targeted therapy

Human Epidermal Growth Factor Receptor 2 (HER2) is one of the well-established therapeutic targets in gastric cancer, with an expression rate of approximately 15%–20%, and is associated with tumor aggressiveness and poor prognosis (56). Trastuzumab combined with chemotherapy remains the current standard first-line treatment for patients with HER2-positive advanced gastric cancer (57). However, the response rate and duration of benefit from this combination therapy remain limited, highlighting the urgent need for novel therapeutic strategies to enhance efficacy. With the advancement of immunotherapy, the combination of ICIs with anti-HER2 targeted therapy has emerged as a promising therapeutic strategy. The KEYNOTE-811 trial evaluated the efficacy of pembrolizumab combined with trastuzumab and standard chemotherapy (FP or CAPOX) in patients with HER2-positive advanced gastric or gastroesophageal junction adenocarcinoma (58). The results demonstrated that the pembrolizumab combination regimen significantly improved the ORR (74.4% vs. 51.9%, p < 0.01), with longer duration of response and favorable safety. This regimen has been approved by the FDA as a first-line treatment option for patients with HER2-positive and PD-L1–positive disease (59). However, clinical data for patients with HER2-positive but PD-L1–negative tumors remain insufficient, and whether they can benefit from ICIs-based combination therapy requires further investigation (60). Current studies have also not yet established whether molecular biomarkers such as TMB or MSI can serve as predictive indicators of immunotherapy response in this patient population (61). The synergistic effects of ICIs and HER2-targeted therapy offer new therapeutic opportunities for patients with HER2-positive advanced gastric cancer; however, defining the optimal patient population and elucidating the underlying mechanisms remain key areas for future research (62).

#### 3.4.1 Clinical implications

- Who benefits/in what context: In HER2-positive advanced gastric/GEJ adenocarcinoma, the strongest current evidence for adding immunotherapy is in HER2-positive, PD-L1–positive disease, where pembrolizumab + trastuzumab + chemotherapy improved ORR in KEYNOTE-811 and has regulatory support as a first-line option.
- Practical interpretation: For eligible patients, PD-1 blockade combined with HER2 targeting can enhance responses on top of the trastuzumab–chemotherapy backbone, providing a rational first-line intensification strategy when PD-L1 is positive.
- What remains uncertain: Evidence remains limited for HER2-positive but PD-L1–negative tumors, and it is unclear whether MSI/TMB meaningfully refine selection in this subgroup; optimal patient selection and mechanisms of synergy/resistance warrant further study.

### 3.5 Combination targeted therapy: new breakthrough in microenvironment modulation

In recent years, combination therapy involving ICIs and anti-angiogenic agents has emerged as a major research focus in the field of gastric cancer immunotherapy. Anti-angiogenic therapy not only exerts direct antitumor effects but also remodels the tumor immune microenvironment, enhances immune cell infiltration and activation, and thereby creates favorable conditions for ICIs, demonstrating a synergistic mechanism of action (63). The LEAP-005 trial evaluated the efficacy of pembrolizumab combined with lenvatinib in various advanced, treatment-refractory solid tumors (64, 65). In the gastric cancer cohort, the combination regimen achieved an ORR of 10% and a disease control rate (DCR) exceeding 60%, with some patients achieving long-term survival. These results demonstrated notable synergistic activity and clinical potential, providing preliminary evidence for the application of ICIs plus anti-angiogenic therapy in gastric cancer (66). Among patients with HER2-negative gastric cancer who had failed prior treatments, the combination achieved disease control in a subset of patients with an acceptable safety profile, laying the groundwork for further clinical validation (67). Moreover, an increasing number of studies are investigating combination strategies of ICIs with other targeted agents, such as FGFR inhibitors (68), claudin 18.2–directed therapies (69), and transforming growth factor-β (TGF-β) inhibitors (70). These approaches, particularly ICIs plus anti-angiogenic therapy, hold the potential to overcome the limitations of ICI monotherapy and offer novel therapeutic options for patients with highly immunoresistant gastric cancer (71).

#### 3.5.1 Clinical implications

- Who benefits/in what context: ICI plus anti-angiogenic combinations are currently best supported in advanced, heavily pretreated (treatment-refractory) gastric cancer—particularly in HER2-negative patients after prior lines—where early studies suggest disease control in a subset.
- Practical interpretation: Anti-angiogenic agents may “prime” the TME for ICIs; clinically, signals are modest in ORR but higher in DCR, so these combinations are primarily being explored in clinical trials and selected later-line settings.
- What remains uncertain: GC-specific prospective validation and biomarker-guided selection are lacking; optimal sequencing and partner targets remain to be defined.

### 3.6 Combination with radiotherapy: an attempt to activate local and systemic immunity

Radiotherapy not only exerts direct cytotoxic effects but also enhances the immune response by inducing tumor cell necrosis, releasing tumor-associated antigens, promoting antigen presentation, and activating both innate and adaptive immunity (72). The combination of radiotherapy with ICIs has shown synergistic effects across various solid tumors, and exploration of this approach in gastric cancer is becoming increasingly active. Gastric cancer has traditionally been considered an “immunologically cold” tumor, characterized by limited T-cell infiltration and low antigenicity (73, 74). Radiotherapy can enhance the initiation of the primary immune response by activating the cGAS–STING pathway and inducing type I interferon release, thereby increasing T-cell infiltration and effector function, which ultimately improves the therapeutic response to ICIs (75, 76). At present, multiple clinical studies are actively investigating this strategy in gastric cancer. The phase II Neo-PLANET study demonstrated that camrelizumab combined with concurrent chemoradiotherapy as a neoadjuvant regimen for locally advanced gastric adenocarcinoma significantly improved pathological response rates with an acceptable safety profile, providing important clinical evidence for perioperative immunotherapy strategies in this patient population (77). The combination of radiotherapy and immunotherapy opens a new avenue for gastric cancer treatment, with the potential to improve local control, reduce distant metastasis, and enhance long-term outcomes in patients with locally advanced disease.

#### 3.6.1 Clinical implications

- Who benefits/in what context: The most plausible clinical niche is locally advanced disease where radiotherapy/CRT is already part of multimodality management, and adding ICIs aims to convert an immunologically “cold” tumor into a more inflamed phenotype.
- Practical interpretation: The mechanistic rationale is that radiotherapy can enhance antigen release/presentation and stimulate cGAS–STING/type I IFN signaling to increase T-cell infiltration and function—supporting radiotherapy as an “immune primer” for ICIs.
- What remains uncertain: Evidence remains early in gastric cancer; confirmatory trials are needed, and optimal dosing/fractionation, sequencing, and patient selection are not established.

### 3.7 Combination of dual ICIs: exploring dual immune checkpoint blockade strategies

To further activate antitumor immune responses and overcome T-cell exhaustion and tumor immune evasion, dual immune checkpoint blockade strategies have attracted increasing attention. Among these, the combination of PD-1/PD-L1 inhibitors with CTLA-4 inhibitors is one of the most extensively investigated approaches. The PD-1/PD-L1 pathway primarily functions in the later stages of T-cell activation by reducing T-cell activity through peripheral immune regulation, whereas the CTLA-4 pathway mainly regulates the early stages of T-cell activation by limiting initial T-cell expansion. Therefore, their combined use can synergistically enhance immune responses at both the activation and effector stages, theoretically providing stronger antitumor activity (31). The CheckMate-032 (78) and CheckMate-649 (25, 46) studies evaluated the efficacy of nivolumab combined with ipilimumab in advanced gastric cancer. The results showed that the ORR in the combination group was significantly higher than that in the monotherapy group, and some patients achieved durable responses. Nevertheless, the incidence of grade ≥3 irAEs increased significantly (approximately 26%), limiting the widespread adoption of this regimen in first-line treatment (42). Beyond CTLA-4, combination blockade strategies targeting novel immune checkpoints such as LAG-3, TIM-3, and T cell immunoreceptor with Ig and ITIM domains (TIGIT) are also entering clinical evaluation. For example, the RELATIVITY-047 study demonstrated the efficacy of PD-1 plus LAG-3 inhibition (nivolumab + relatlimab) in melanoma, providing a rationale for expanding this approach to gastric cancer (79). Although multi-ICIs combination therapy offers theoretical advantages for synergistically enhancing immune responses and has shown early signs of efficacy, it still faces significant challenges, including a high incidence of irAEs and a broad toxicity profile, highlighting the urgent need for optimized toxicity management strategies (80).

#### 3.7.1 Clinical implications

- Who benefits/in what context: Dual-checkpoint blockade (PD-1/PD-L1 plus CTLA-4) is biologically rational because it can enhance T-cell priming and effector activity at complementary phases of the immune response; in advanced gastric cancer, early clinical experience suggests higher response signals versus monotherapy in selected patients.
- Practical interpretation: Despite encouraging activity, nivolumab + ipilimumab–based regimens are not broadly adopted as routine first-line options in gastric cancer because toxicity is a major barrier—grade ≥3 irAEs have been reported at ~26% in cited studies—so use should remain within carefully selected patients and/or clinical trials with robust toxicity monitoring and management.
- What remains uncertain: The therapeutic index remains the key challenge—optimal combinations, biomarker selection, and dosing strategies to reduce irAEs while preserving efficacy are still undefined; newer checkpoints (e.g., LAG-3/TIGIT) are under evaluation.

Currently, strategies for ICI therapy in gastric cancer are continuously evolving, and future research should focus on developing individualized immunotherapy approaches to deliver more precise and effective treatment options for patients.

Summary of pivotal clinical studies evaluating ICIs across various treatment settings, including second-line and later-line monotherapy, first-line combination with chemotherapy, targeted or dual checkpoint blockade, perioperative therapy, and novel combinations. Outcomes demonstrate the evolving role of ICIs in improving objective response rate (ORR), progression-free survival (PFS), overall survival (OS), and disease-free survival (DFS). Ongoing phase II/III trials continue to explore perioperative and combination strategies to optimize efficacy, expand indications, and integrate immunotherapy into multimodal treatment paradigms.

## 4 Challenges in ICI therapy

Although ICIs have achieved significant progress in the treatment of gastric cancer, their clinical application still faces multiple challenges, including resistance mechanisms, irAEs, insufficient biomarkers, and an immunosuppressive TME.

### 4.1 Mechanisms of resistance

The antitumor efficacy of ICIs in gastric cancer is limited by both primary and acquired resistance, which interact to significantly reduce the durability and effectiveness of treatment.

#### 4.1.1 Primary resistance

Primary resistance refers to the lack of therapeutic response when patients first receive ICIs treatment, typically manifested as no significant changes in tumor biomarkers, no evident radiological tumor shrinkage, and no improvement in PFS (81, 82). It is closely associated with low immune checkpoint expression on tumor cells, TMB and microsatellite status, the immunosuppressive TME, defective antigen presentation, and aberrant signaling pathways (83, 84).

PD-L1 is currently the most widely used predictive biomarker, and its expression level directly influences the efficacy of PD-1/PD-L1 blockade. In some gastric cancer patients, PD-L1 expression is low (CPS < 1 or < 5) or completely absent, limiting the ability of ICIs to disrupt immunosuppressive signaling and leading to primary resistance (85). PD-L1 expression is regulated by multiple mechanisms, including intrinsic genetic alterations, epigenetic modifications, and induction by proinflammatory factors within the TME. Abnormalities in signaling pathways such as JAK/STAT (81) and NF-κB (86) can disrupt PD-L1 expression and reduce sensitivity to immunotherapy. In addition to PD-L1, low expression of other immune checkpoint molecules such as PD-1 and CTLA-4 can also impair T-cell function and diminish the overall efficacy of ICIs (87). Patients with MSI-H or TMB-H are more sensitive to ICI therapy, because they harbor abundant neoantigens that enhance T-cell recognition and activation, thereby triggering a stronger antitumor immune response (88). However, most gastric cancer patients are microsatellite-stable (MSS) or have low TMB, resulting in insufficient antigenicity, inefficient antigen presentation, limited T-cell activation, and poor responsiveness to ICIs (89).

The TME of gastric cancer is often enriched with immunosuppressive cells such as Tregs, myeloid-derived suppressor cells (MDSCs), and tumor-associated macrophages (TAMs), which collaboratively exert immunosuppressive effects (90). Tregs secrete immunosuppressive cytokines such as interleukin-10 (IL-10) and TGF-β, which inhibit the proliferation and cytotoxic function of CD8^+^ effector T cells (91). MDSCs suppress T-cell expansion by releasing reactive oxygen species (ROS) and reactive nitrogen species (RNS), working synergistically with Tregs to establish an immunotolerant environment (92). TAMs in gastric cancer frequently exhibit immunosuppressive and pro-tumor functional programs—including IL-10/TGF-β production, pro-angiogenic signaling (e.g., VEGF), and tissue-remodeling activities—reflecting a heterogeneous and dynamic spectrum rather than a rigid M1/M2 dichotomy. These TAM programs reinforce immune suppression by dampening effector T-cell function while concurrently promoting angiogenesis, tumor growth, invasion, and metastatic dissemination (93).

Loss or mutation of major histocompatibility complex (MHC) class I molecules in tumor cells is another critical factor affecting ICIs efficacy (94). MHC-I plays a crucial role in antigen presentation, and its loss allows tumor cells to evade immune surveillance, reducing the antitumor activity of ICIs (95). Studies have shown that mutations in the β2-microglobulin (β2M) gene can lead to loss of MHC-I expression, impairing CD8^+^ T-cell activation and tumor antigen recognition (96, 97). In addition, some gastric cancer patients exhibit reduced or lost expression of tumor antigens, making it difficult for T cells to recognize and target tumor cells (80).

Aberrant signaling pathways are another key mechanism underlying tumor resistance to ICIs. Mutations in JAK1/2 can inactivate the JAK/STAT signaling pathway, weakening Interferon-γ (IFN-γ)–mediated immune responses and inducing resistance to ICIs (98). Aberrant activation of the Wnt/β-catenin pathway can inhibit dendritic cell (DC) infiltration and impede CD8^+^ T-cell activation (99). Moreover, abnormal activation of the MAPK and PI3K/AKT signaling pathways can promote the secretion of immunosuppressive factors, driving the TME toward an immunotolerant state (100).

#### 4.1.2 Acquired resistance

Acquired resistance refers to the phenomenon in which tumors, after initially responding to ICI therapy, gradually adapt under continuous immune pressure, leading to diminished efficacy and eventual disease progression or recurrence (101). It is associated with changes in immune checkpoint expression, tumor heterogeneity, T-cell dysfunction, and the activation of immune evasion mechanisms (89, 102).

During ICIs treatment, immune checkpoint signaling undergoes dynamic remodeling. Some tumor cells may downregulate or lose PD-L1 expression, thereby weakening the capacity of PD-1/PD-L1 blockade to interrupt immunosuppressive signaling (103–105). In parallel, tumor cells and immunosuppressive immune subsets can upregulate alternative checkpoints, including LAG-3, TIM-3, TIGIT, and V-domain Ig suppressor of T cell activation (VISTA), creating compensatory inhibitory pathways that further restrain T-cell activity. For example, LAG-3 binding to MHC-II inhibits T-cell activation (80), TIM-3 interaction with Galectin-9 promotes T-cell exhaustion (102), and TIGIT competes with CD226 (DNAM-1) signaling to impair cytotoxic function in T cells and NK cells (106). The coordinated upregulation of these pathways can enable immune escape from PD-1/PD-L1 or CTLA-4 blockade alone, ultimately driving therapeutic resistance (107).

Meanwhile, tumors may evolve resistant subclones via clonal selection, genomic instability, and epigenetic remodeling (108). Under immune pressure, ICIs preferentially eliminate immunogenic or immune-sensitive clones, whereas variants with stronger immune-evasion capacity survive and expand. Genetic mutations and epigenetic alterations can further increase intratumoral heterogeneity and accelerate resistance development (109). Epigenetic remodeling, such as PD-L1 promoter methylation and chromatin reorganization, may dynamically regulate immune-related genes, facilitating continuous adaptation during therapy (110). Spatial heterogeneity also contributes: distinct tumor regions can differ in PD-L1 expression, antigen presentation capacity, and immunosuppressive mediator secretion, creating “immune-privileged” niches within the TME that sustain escape under treatment (111).

T-cell dysfunction is another central feature of acquired resistance, primarily characterized by exhaustion and metabolic reprogramming (112, 113). Exhausted T cells show reduced proliferative capacity, diminished effector function, decreased secretion of IFN-γ, TNF-α, and IL-2 (114), and persistent high expression of co-inhibitory receptors such as TIM-3, LAG-3, TIGIT, and VISTA (115). Under prolonged antigen stimulation, cytotoxic activity progressively declines, weakening tumor control. In addition, nutrient competition within the TME—particularly glucose competition between tumor cells and T cells—impairs T-cell bioenergetics and forces T cells into a low-energy state (116, 117). Increased lactate accumulation further suppresses T-cell activation and reinforces an immunosuppressive milieu (118). Collectively, these metabolic constraints compromise sustained antitumor immunity and facilitate the establishment of immune-evasion programs during continued ICIs exposure (119).

**Figure 2:**
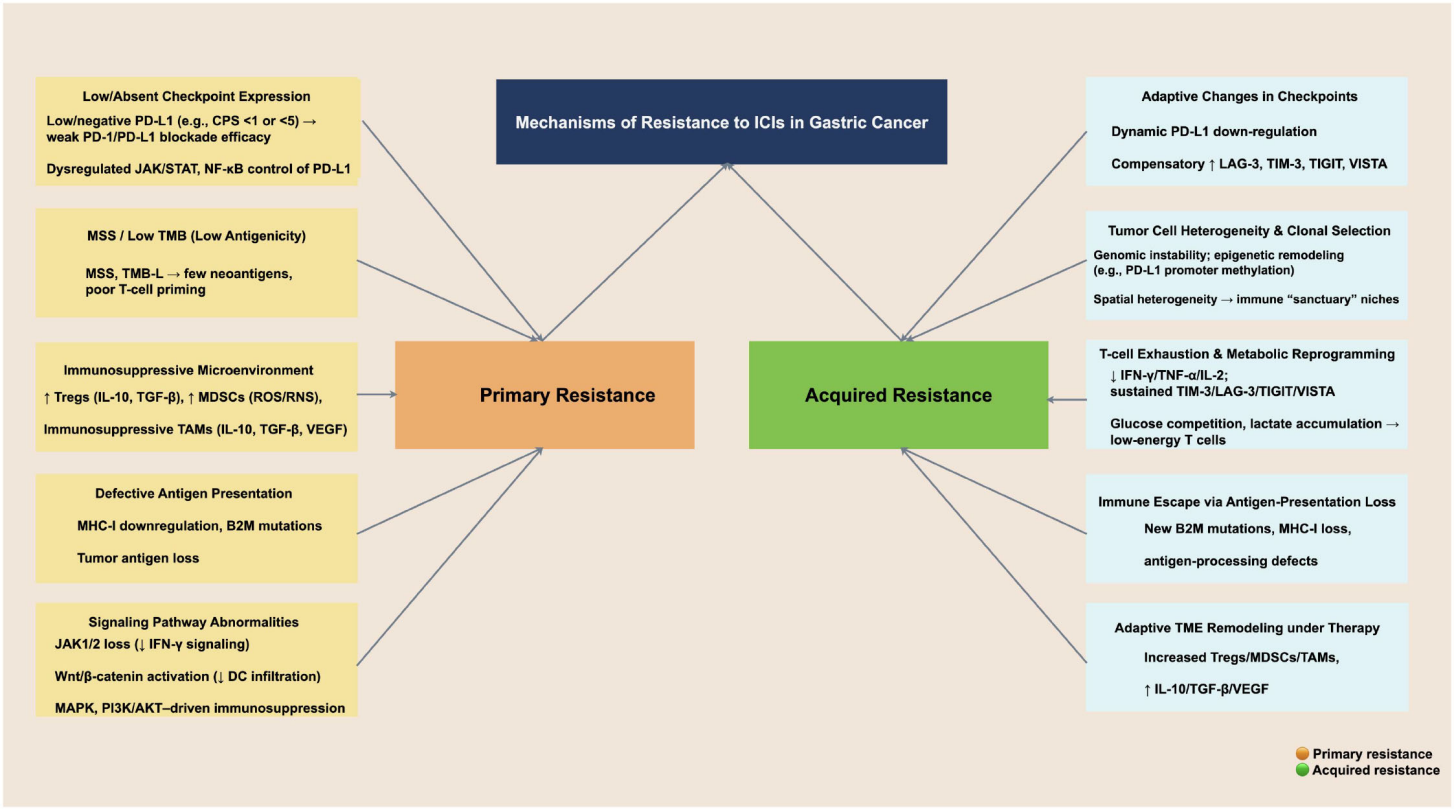
Mechanisms of resistance to ICIs in gastric cancer. Resistance involves two main mechanisms: primary resistance, characterized by low checkpoint expression, MSS/low TMB, immunosuppressive TME, antigen-presentation defects, and signaling pathway alterations. Acquired resistance, including adaptive checkpoint changes, tumor cell heterogeneity, T-cell dysfunction, immune escape, and therapy-induced TME remodeling.

Taken together, acquired resistance reflects the convergent effects of adaptive checkpoint remodeling, clonal evolution and heterogeneity, and progressive T-cell dysfunction within a therapy-pressured and increasingly immunosuppressive TME, culminating in immune escape and loss of ICIs-mediated tumor control (120, 121) (**Figure 2**).

### 4.2 Immune-related adverse events

irAEs are one of the major challenges during ICI therapy, significantly affecting treatment continuity and patients’ quality of life. The underlying mechanisms of irAEs have not been fully elucidated, but they are generally believed to arise from abnormal activation of the host’s autoimmune response. While ICIs relieve immune suppression and activate T cell–mediated immune responses, they may also trigger excessive immune activation, disrupt immune tolerance (79), and provoke aberrant autoimmune reactions, leading to multi-organ immune attacks and the development of irAEs (122). The occurrence of irAEs may also be associated with the generation of autoantibodies, excessive release of proinflammatory cytokines (e.g., IFN-γ, IL-6, TNF-α), functional imbalance of immune cell subsets, and gut microbiota dysbiosis (123). Genetic susceptibility, a history of autoimmune disease, and combination immunotherapy regimens may further increase the risk of irAEs (124).

irAEs are highly heterogeneous and can affect multiple organ systems, including the skin, gastrointestinal tract, lungs, liver, endocrine, cardiovascular, and nervous systems. Common manifestations include rash, pruritus, thyroid dysfunction, colitis, pneumonitis, elevated liver enzymes, and myocarditis (125, 126). Immune-related cardiac toxicity, hepatotoxicity, and other adverse effects have also been reported (127). In some cases, multiple organs are simultaneously affected, leading to treatment discontinuation and life-threatening complications (128).

Clinical data indicate that the incidence and severity of irAEs vary across different ICIs and treatment regimens. The ATTRACTION-2 study reported an overall irAE incidence of ~10% in gastric cancer patients treated with nivolumab, with most being grade 1–2 and grade 3–4 irAEs occurring in ~4% (129, 130). In the KEYNOTE-059 trial, pembrolizumab-induced irAEs occurred in approximately 17–20% of patients, with grade 3–5 events in ~3–4% (43). Additionally, the CheckMate-649 trial reported a high incidence (~60%) of grade ≥3 treatment-related adverse events (TRAEs) with nivolumab plus chemotherapy, with irAEs occurring in ~5–10% of patients (46), suggesting an increased risk of immune toxicity with combination regimens.

Although most irAEs can be managed with immunosuppressive therapy (e.g., corticosteroids), severe events may lead to treatment discontinuation or permanent withdrawal, ultimately compromising long-term survival benefits (131, 132). Patients with gastric cancer often have poor nutritional status, heavy tumor burden, and impaired gastrointestinal function, all of which can exacerbate irAEs and complicate their clinical management. The unpredictability and interindividual variability of irAEs pose significant challenges (133), and no widely accepted, highly sensitive, and specific predictive biomarkers currently exist. Efforts have been made to predict irAEs risk by analyzing genetic polymorphisms, serum cytokine levels, autoantibody profiles, peripheral immune cell composition, and gut microbiota characteristics, but no standardized predictive model has yet been established (134). Some studies have also suggested a possible association between the occurrence of irAEs and therapeutic efficacy, as patients who develop irAEs often exhibit higher ORR and longer survival (135). However, this correlation has yet to be fully validated by large-scale clinical data.

**Figure 3:**
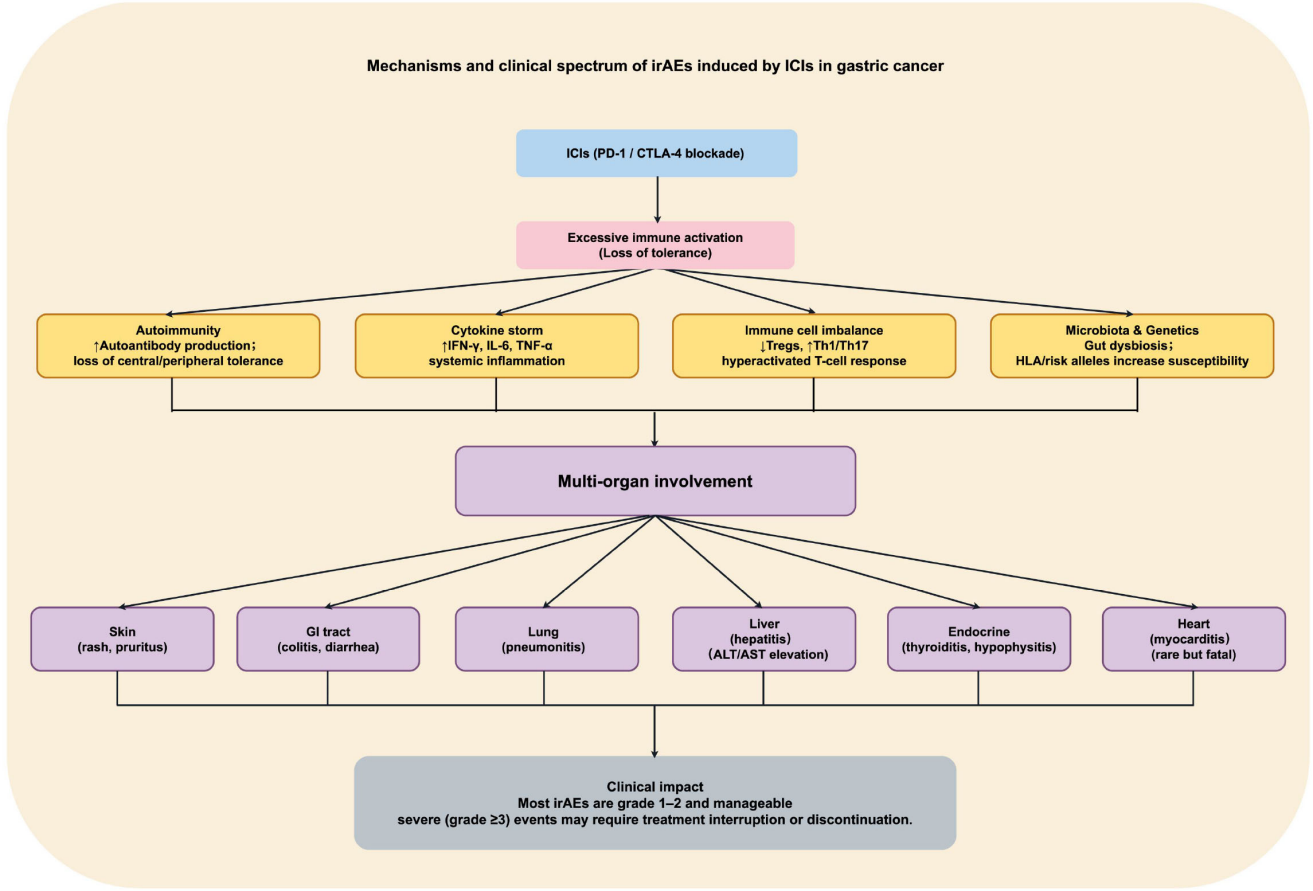
Mechanisms and clinical spectrum of irAEs induced by ICIs. Loss of immune tolerance following PD-1/CTLA-4 blockade can result in autoimmunity, cytokine storms, T-cell dysregulation, and microbiota–genetic susceptibility, leading to multi-organ irAEs such as skin rash, colitis, pneumonitis, hepatitis, thyroiditis, and myocarditis. Most events are mild and manageable, but severe cases may necessitate treatment interruption.

Standardized management of irAEs is essential to ensure the safety and continuity of ICI therapy. Clinical guidelines recommend a comprehensive baseline assessment prior to treatment — including liver, kidney, thyroid, and pulmonary function, as well as autoimmune history — and continuous monitoring during therapy. Once irAEs occur, appropriate interventions should be implemented according to severity. Mild irAEs can often be managed symptomatically without interrupting treatment, whereas severe cases require immediate discontinuation and administration of corticosteroids or other immunosuppressants (e.g., tocilizumab, infliximab, cyclosporine A) (136, 137). For unresolved irAEs, permanent discontinuation of immunotherapy should be considered. Future efforts should focus on elucidating the immunological basis of irAEs, developing predictive models and personalized intervention strategies, establishing early-warning systems, and optimizing comprehensive management to improve the safety of ICIs and enhance patients’ quality of life (138) (**Figure 3**).

### 4.3 Insufficient biomarkers

Although ICIs have demonstrated efficacy across various malignancies, accurately identifying responsive subgroups remains a key limitation to their broader application in gastric cancer. In recent years, several biomarkers—including PD-L1, CTLA-4, MSI-H, deficient mismatch repair (dMMR), and TMB—have been introduced into clinical practice (139–141). However, their predictive value in gastric cancer is limited and requires further validation for routine applicability.

PD-L1 is currently the most widely used predictor of ICIs efficacy and is commonly assessed via the combined positive score (CPS). The KEYNOTE-059 and KEYNOTE-061 trials indicated greater benefit from pembrolizumab in patients with CPS ≥1 or ≥10 (43–45). Nevertheless, PD-L1 expression is subject to spatial and temporal heterogeneity, with variable results across tumor sites, sampling methods, and assay platforms (142). Moreover, some PD-L1–negative or low-expressing patients still respond to ICIs (143, 144). Abundant stromal and mucinous components in gastric tumors may compromise assay accuracy, further limiting clinical utility.

CTLA-4 is expressed mainly on activated T cells and inhibits proliferation and activity by competitively binding CD80/CD86 and blocking CD28-mediated co-stimulation, thereby promoting immune tolerance (145). While CTLA-4 inhibitors (e.g., ipilimumab) show promise in combination immunotherapy for melanoma and non–small cell lung cancer (NSCLC), their value in gastric cancer remains uncertain (146). Although CTLA-4 expression is often elevated in gastric tumors and correlates with an immunosuppressive microenvironment, definitive evidence that high CTLA-4 expression predicts ICIs benefit is lacking (147). Moreover, CheckMate-032 and CheckMate-649 indicate that adding CTLA-4 blockade to PD-1/PD-L1 inhibition enhances immune responses but significantly increases irAEs, limiting broader use (46, 78).

MSI-H—widespread microsatellite instability from replication errors—is a validated sensitivity marker in gastric cancer (148), associated with higher response rates and durable control under ICI therapy (149). However, MSI-H comprises only ~5%–10% of gastric cancers, is more frequent in Western than Asian populations, and is enriched in early-stage, mucosa- or superficially invasive tumors (150). MSI-H tumors also exhibit characteristic clinicopathologic features—older age, female sex, distal location, and intestinal histology—which constrain broad applicability (151). With disease progression, MSI-H prevalence declines in advanced or metastatic disease, diminishing its predictive value in late-stage patients.

dMMR refers to mutations or loss of expression in key MMR genes (MLH1, MSH2, MSH6, PMS2), leading to ineffective correction of replication errors (152). dMMR is highly correlated with MSI-H but not entirely concordant (153). dMMR tumors are more sensitive to ICIs and can achieve significant responses and survival benefits (88). However, dMMR is also uncommon in gastric cancer; most tumors are pMMR and respond poorly to ICIs, limiting clinical benefit (154, 155). In addition, testing methods and criteria for dMMR remain non-uniform, introducing technical variability and interpretive bias (156).

TMB, defined as the number of nonsynonymous mutations per genome unit, is frequently used as a crude surrogate of tumor ‘foreignness’ and may correlate with response to ICIs. However, not all mutations contribute equally to immunogenicity: only a subset generate neoantigens that are sufficiently expressed, properly processed, and effectively presented via MHC class I molecules to be recognized by CD8^+^ T cells. Consequently, the quality and presentability of mutation-associated neoantigens—and tumor-intrinsic defects in antigen processing/presentation—can decouple high TMB from clinical benefit (157, 158). Moreover, mutation clonality appears highly relevant: clonal mutations (and the resulting clonal neoantigens) may provide more stable immune targets than subclonal alterations, particularly under the evolutionary bottleneck imposed by immunotherapy. Recent perspectives further suggest that clonal alterations persisting through immune selection—such as those occurring in haploid genomic regions or present in multi-copy states—may carry stronger predictive value for ICI response than bulk TMB alone (159). Nevertheless, in gastric cancer, overall TMB is generally low and TMB-high cases are uncommon; additionally, lack of assay standardization across platforms, panels, and thresholds impairs inter-center comparability (160). Importantly, MSI-associated hypermutation represents one of several mutational processes that can elevate TMB, rather than a simple overlap between the two. Other contributors include POLE/POLD1 alterations (ultramutated phenotype), APOBEC-driven mutagenesis, mutagen-associated genomic signatures, and additional endogenous mutational processes. Therefore, the biological basis of ‘TMB-high’ can be heterogeneous across patients, which complicates interpretation of TMB as an independent predictor of ICI benefit in gastric cancer (161).

**Figure 4:**
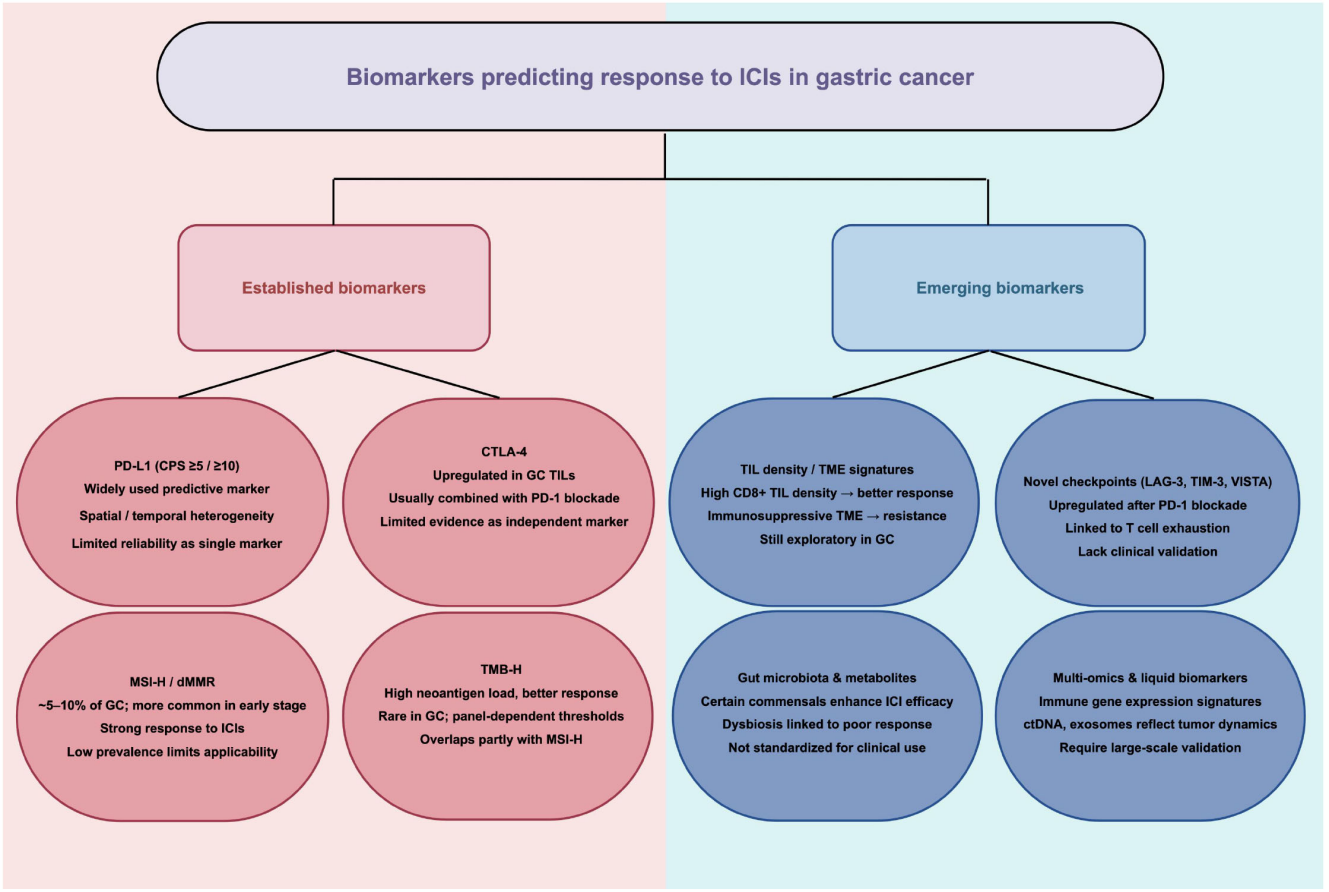
Biomarkers predicting response to ICIs in gastric cancer. Established biomarkers, including PD-L1, CTLA-4, MSI-H/dMMR, and TMB-H, are clinically validated predictors of ICIs efficacy. Emerging biomarkers, such as TIL density, novel checkpoints, microbiota profiles, and multi-omics markers, provide additional predictive insights but remain largely exploratory and require further clinical validation.

Beyond these markers, emerging predictors—TME features (immunosuppressive mediators), novel checkpoints (LAG-3, TIM-3, VISTA), gut microbiota composition, tumor metabolites, multi-omics signatures, and circulating biomarkers—are under active investigation (162–166). However, most evidence remains exploratory, with limited large-scale validation and no standardized testing frameworks. Currently, gastric cancer immunotherapy lacks an efficient, stable predictive system. Single biomarkers cannot capture the complexity and dynamism of the immune microenvironment, and their performance does not yet meet clinical precision-selection requirements (17). Future research should prioritize clinically actionable markers and develop multidimensional, multi-omics composite prediction models to enable precise and individualized immunotherapy in gastric cancer (167) (**Figure 4**).

### 4.4 Tumor microenvironment suppression

**Figure 5:**
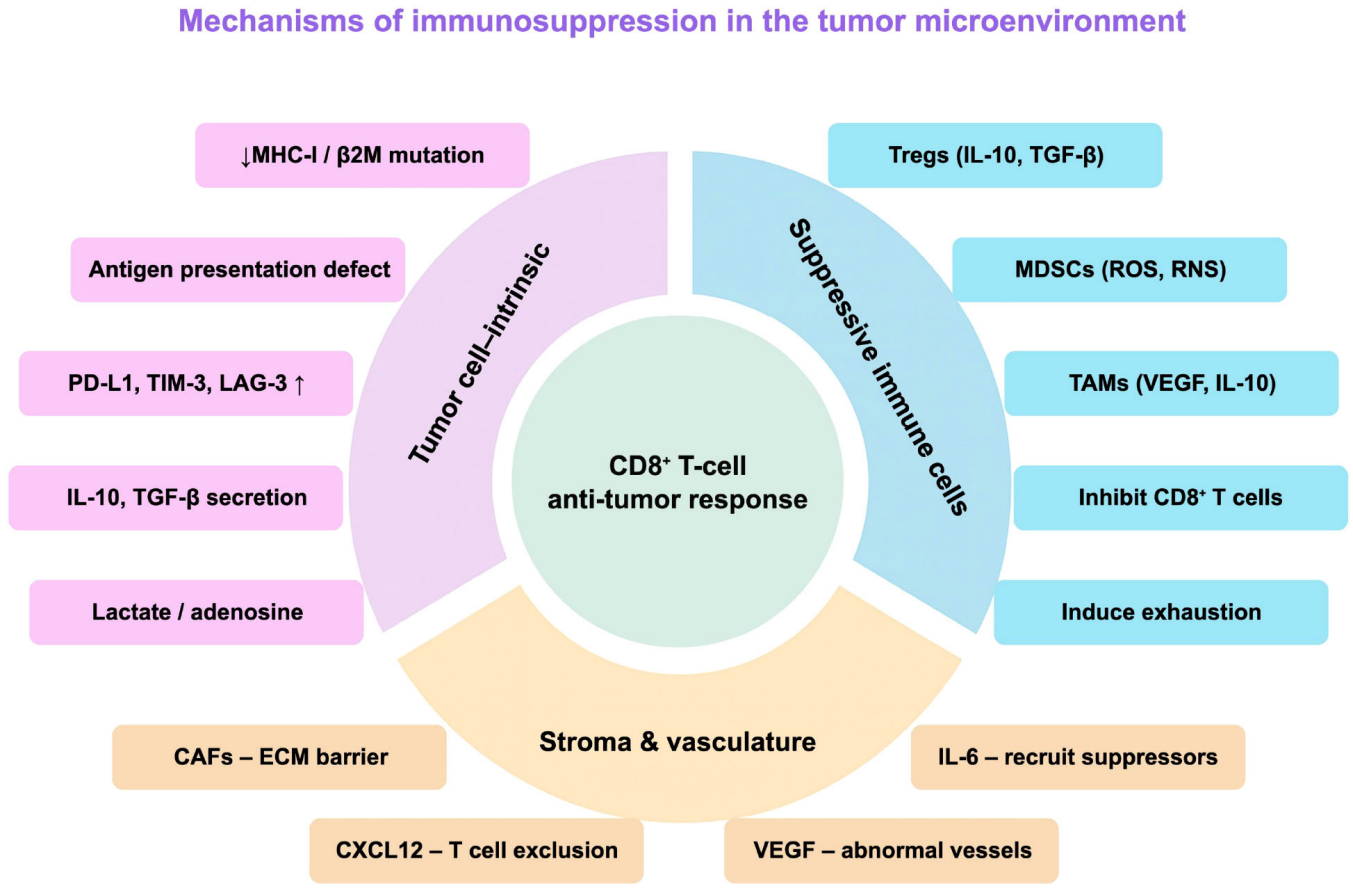
Mechanisms of immunosuppression in the TME. Multiple factors suppress the CD8^+^ T-cell anti-tumor response in gastric cancer. Tumor-intrinsic mechanisms include MHC-I or β2M loss, antigen presentation defects, checkpoint upregulation, cytokine secretion, and metabolic suppression. Suppressive immune cells (Tregs, MDSCs, immunosuppressive TAMs) and stromal or vascular factors (CAFs, CXCL12, IL-6, VEGF) further inhibit T cells, induce exhaustion, and create barriers that sustain an immunosuppressive TME.

TME is a critical determinant of the therapeutic efficacy of ICIs, consisting of tumor cells, immune cells, stromal cells, and a variety of molecular components (**Figure 5**).

#### 4.4.1 Tumor cells

Tumor cells employ multiple mechanisms to shape an immunosuppressive microenvironment, impairing T-cell activation, proliferation, and effector functions, thereby directly diminishing the antitumor efficacy of ICIs. Immunosuppressive cytokines secreted by tumor cells, such as TGF-β and IL-10, not only hinder the activation of effector T cells but also promote the recruitment and expansion of immunosuppressive cells including Tregs, MDSCs, and TAMs, thereby reinforcing the immunosuppressive characteristics of the TME (168). Metabolic dysregulation in tumor cells leads to the accumulation of byproducts such as lactate and adenosine, which further suppress T-cell function and attenuate antitumor immune responses (169). In addition, tumor cells can disrupt antigen presentation pathways through specific genetic or epigenetic mechanisms, thereby evading immune recognition. For example, downregulation or loss of MHC-I expression markedly reduces T-cell recognition of tumor antigens, enabling tumor cells to escape immune surveillance (94). Mutations in the β2M gene, as well as functional defects in antigen-processing and presentation molecules, can impair antigen presentation and facilitate tumor immune evasion (170). Moreover, certain tumors upregulate additional inhibitory receptors (such as TIGIT, LAG-3, and TIM-3) or their ligands, further suppressing T-cell activity (171).

#### 4.4.2 Immune cells

Immune cells play a pivotal role in tumor immune evasion, where suppression of effector T-cell function and the accumulation of immunosuppressive cells are primary factors limiting the efficacy of ICIs. CD8^+^ T cells, as the principal effector cells in ICI therapy, are responsible for recognizing and eliminating tumor cells. However, in the TME of gastric cancer, the activity of CD8^+^ T cells is impaired by various immunosuppressive factors (168). Tregs not only secrete immunosuppressive cytokines such as IL-10 and TGF-β to directly inhibit the proliferation and cytotoxic activity of CD8^+^ T cells, but also suppress the antigen-presenting capacity of DCs (10, 172). High infiltration of Tregs in gastric cancer tissues is strongly associated with poor ICIs efficacy and unfavorable prognosis. MDSCs secrete molecules such as ROS and RNS to inhibit effector T-cell proliferation, induce their functional exhaustion, and promote Tregs expansion, thereby establishing an immunosuppressive positive feedback loop that further reinforces the immunosuppressive state of the TME (173). Immunosuppressive TAMs constitute a major suppressive myeloid population in the TME and display a spectrum of pro-tumor functional states. By secreting mediators such as VEGF and IL-10 (often together with TGF-β, arginase-1–related pathways, and other suppressive signals), these TAM programs can dampen CD8^+^ T-cell activation and effector function, limit antigen presentation, and reinforce immune tolerance. In parallel, TAM-derived pro-angiogenic factors promote abnormal neovascularization and vascular dysfunction, which further restricts immune-cell trafficking into the tumor core. Moreover, through secretion of matrix-remodeling enzymes and growth factors, immunosuppressive TAMs contribute to stromal remodeling and extracellular-matrix reorganization, thereby facilitating tumor invasion and metastatic dissemination. Collectively, these functional TAM states help maintain an immunosuppressive, immune-excluded microenvironment that undermines the efficacy of ICIs in gastric cancer (174).

#### 4.4.3 Stromal cells

Stromal cells within the TME not only provide structural support for tumor growth but also secrete a variety of cytokines that regulate immune cell activity and influence tumor cell sensitivity to ICI therapy. Among them, fibroblasts secrete large amounts of extracellular matrix (ECM) components, such as collagen and fibronectin, which remodel the TME and establish a dense physical barrier that prevents effector T-cell infiltration into the tumor core. Fibroblasts also secrete immunosuppressive factors such as TGF-β, IL-6, and C-X-C motif chemokine ligand 12 (CXCL12) (175), which not only inhibit the activation and cytotoxic function of effector T cells but also promote the recruitment and expansion of immunosuppressive cells, thereby exacerbating the immunosuppressive state (176). Persistent overexpression of chemokines such as CXCL12 further impedes T-cell infiltration into the tumor core, resulting in a “T-cell exclusion” phenotype (177). Vascular endothelial cells secrete high levels of VEGF, inducing the formation of structurally abnormal and functionally defective blood vessels, which restrict T-cell entry into the tumor core and create an “immune-excluded” microenvironment (178). Sustained overexpression of VEGF not only suppresses T-cell activation and effector function but also facilitates the upregulation of immune checkpoint molecules, thereby synergistically promoting tumor immune evasion (179).

Tumor cells, immune cells, and stromal cells interact through cytokines, metabolic byproducts, and signaling pathways to establish a complex immunosuppressive network that substantially impairs the efficacy of ICIs. Elucidating these mechanisms in greater depth will provide a theoretical foundation for optimizing immunotherapeutic strategies, developing combinatorial interventions, and identifying novel therapeutic targets.

## 5 Future research directions

ICIs have opened a new therapeutic avenue for gastric cancer, significantly improving survival outcomes in a subset of patients. However, their efficacy exhibits marked interindividual variability, and their widespread clinical application remains constrained by resistance mechanisms, insufficient predictive biomarkers, and immune-related toxicities. Therefore, future research must focus on optimizing therapeutic strategies, exploring the synergistic potential of combination regimens, and developing multidimensional individualized interventions to accelerate the clinical translation of precision immunotherapy, ultimately enhancing the durability of efficacy and patient quality of life.

### 5.1 Research and application of biomarkers

The therapeutic efficacy of ICIs varies considerably among individuals, with only a fraction of patients achieving durable benefit (180). Hence, there is an urgent need to establish reliable biomarker systems to predict efficacy, guide patient selection, and monitor treatment response. Although traditional biomarkers such as PD-L1, CTLA-4, MSI-H, and TMB have shown promise, their predictive value remains insufficient for precise clinical stratification. Single traditional biomarkers are inadequate for comprehensive prediction of immunotherapy outcomes (181). Future studies should prioritize the discovery and validation of more accurate and reliable biomarkers to improve the identification of patients.

The novel immune checkpoints provide new opportunities to overcome current limitations. Molecules such as LAG-3, TIM-3, and VISTA, which are implicated in T-cell exhaustion and immune evasion, exert regulatory effects in multiple solid tumors (182–184). In gastric cancer, their expression is closely linked to the immunosuppressive TME, patient prognosis, and response to ICIs. For instance, LAG-3 is frequently co-expressed with PD-1, suggesting synergistic immunosuppressive activity and potential as a target for dual checkpoint blockade (185).

Tumor-infiltrating lymphocytes (TILs) recognize and eliminate target cells expressing tumor-associated antigens. Studies have demonstrated that high TIL abundance, particularly CD8^+^ T-cell infiltration, is generally associated with favorable prognosis and stronger immune responsiveness (186). In gastric cancer, TIL levels have been shown to correlate closely with ICIs efficacy, with patients exhibiting high TIL expression often experiencing more pronounced clinical benefit (187). Although TILs represent a highly promising predictive biomarker, their mechanisms of action remain incompletely understood, and the lack of standardized detection and evaluation systems hampers their widespread clinical application (188).

The expression profiles of immune-regulatory factors are also emerging as potential predictive biomarkers. Rather than reiterating the mechanistic roles of suppressive immune subsets in resistance (discussed in Sections 4.1 and 4.4), recent studies highlight that the abundance, phenotype/function, and spatial distribution of MDSCs and Tregs may capture the degree of immunosuppression and help stratify patients for ICIs (189). In parallel, under persistent antigenic stimulation, CD8^+^ T cells can adopt an exhausted state (Tex), characterized by high expression of inhibitory receptors such as PD-1, LAG-3, and TIM-3, accompanied by restricted effector activity and diminished proliferative potential (190). Importantly, the extent and quality of Tex programs—including their functional state and spatial organization within the TME—have been linked to ICIs efficacy, supporting their potential utility as predictive biomarkers (191, 192).

Metabolic reprogramming of tumor cells further shapes the immune microenvironment. Lactate, a major product of glycolysis, acidifies the TME, suppresses T-cell activation and effector function, and drives macrophages toward immunosuppressive TAM programs—characterized by increased production of mediators such as IL-10 and TGF-β, pro-angiogenic signaling, and tissue-remodeling activities—thereby reinforcing immune tolerance and undermining the efficacy of ICIs (193). Within the tryptophan metabolic pathway, indoleamine 2,3-dioxygenase (IDO) depletes tryptophan and accumulates kynurenine, which inhibits effector T-cell function and induces immune tolerance (194). The abundance of these metabolites is negatively correlated with ICIs response, suggesting their potential as therapeutic targets. In addition, proinflammatory cytokines such as IL-6 and IL-8 not only regulate stromal remodeling and angiogenesis but also activate immunosuppressive cells through JAK/STAT and NF-κB signaling, thereby impairing T-cell recruitment and activation (195). Immunosuppressive factors such as TGF-β and VEGF are also strongly correlated with ICIs efficacy, making them promising candidates for biomarker development and combinatorial targeting (196, 197).

The gut microbiota also critically modulates the outcomes of immunotherapy. Beneficial strains including Akkermansia muciniphila (198), Bifidobacterium (199), and Faecalibacterium (200) enhance antigen presentation and promote CD8^+^ T-cell activation. Conversely, dysbiosis may foster an immunosuppressive environment, reducing therapeutic sensitivity. Overgrowth of opportunistic pathogens, such as Escherichia coli and Clostridium difficile, has been associated with elevated Treg frequencies and attenuated immune responses, ultimately undermining responsiveness to immunotherapy (201). Evidence from melanoma and NSCLC indicates that modulation of gut microbiota can markedly improve immunotherapeutic outcomes (202). Although data in gastric cancer remain limited, early evidence suggests that gut microbiota can serve as an emerging biomarker for predicting the efficacy of ICIs (203).

Circulating biomarkers, as a non-invasive detection modality, hold significant promise for predicting therapeutic efficacy and dynamically monitoring responses in cancer immunotherapy. Compared with tissue biopsies, they offer advantages of convenient sampling, high reproducibility, and longitudinal tracking, making them particularly suitable for malignancies such as gastric cancer, where tissue acquisition is challenging and intratumoral heterogeneity is pronounced (204). Circulating tumor DNA (ctDNA), released into peripheral blood during tumor cell apoptosis or necrosis, carries mutation profiles that reflect tumor molecular features, while quantitative changes provide an indicator of tumor burden and therapeutic response. A significant decline in ctDNA levels typically indicates therapeutic efficacy, whereas persistent elevation may suggest disease progression or the emergence of resistance, underscoring its clinical value in efficacy monitoring (205). Exosomes, which carry proteins, lipids, DNA, and diverse RNAs (e.g., miRNAs and lncRNAs), serve as crucial mediators of communication between tumors and the immune system (206). Tumor-derived exosomes not only contribute to immune evasion but also mirror the immune status of the TME through their cargo of immunomodulatory factors. Immunoregulatory miRNAs or protein markers within exosomes represent potential tools for predicting immunotherapy responses (207, 208).

The rapid development of emerging technologies such as single-cell RNA sequencing (scRNA-seq), spatial transcriptomics, and multi-omics integration has provided powerful tools for discovering novel biomarkers associated with immunotherapy (209, 210). scRNA-seq enables the characterization of cellular heterogeneity within tumors and their microenvironment, identifying critical immune cell subsets and functional states (211). Single-cell analyses of TILs and TAMs have revealed novel biomarkers linked to immune response intensity and immunosuppressive states, including specific T-cell exhaustion markers and chemokine–receptor expression profiles (211). Spatial transcriptomics integrates gene expression with tissue architecture, capturing immune interactions among neighboring cells and overcoming the spatial limitations of single-cell data (212). Multi-omics integration, encompassing genomic, transcriptomic, epigenomic, proteomic, and metabolomic data, allows systemic and dynamic mapping of regulatory networks in the TME, facilitating the discovery of composite biomarkers (213). With the expanding application of artificial intelligence (AI) and machine learning (ML), the precision and efficiency of biomarker discovery are expected to further improve.

Imaging biomarkers, as non-invasive and highly reproducible tools, enable dynamic monitoring of tumor morphology, metabolism, and TME without tissue sampling, thereby offering superior clinical accessibility and real-time applicability (214, 215). Radiomics applies high-throughput analysis to CT, MRI, and PET images, extracting multidimensional features such as tumor texture, morphology, margin clarity, and density distribution. When integrated with AI and ML, these features can predict immune status, immune cell infiltration, and PD-L1 expression (216, 217). Compared with conventional RECIST criteria, imaging biomarkers can capture immune responses earlier, distinguish pseudoprogression or mixed responses, and guide timely clinical treatment adjustments (218).

Research on immunotherapy-related biomarkers in gastric cancer remains exploratory, and the predictive sensitivity and specificity of single biomarkers are limited. Future investigations should prioritize the following directions: (1) identification and validation of novel immune checkpoints, with development of predictive models and multi-target inhibitory strategies; (2) evaluation of the predictive value of TILs and their subsets in large patient cohorts; (3) mapping of immune, metabolic, and inflammatory networks within the TME using single-cell and spatial transcriptomic technologies to identify novel biomarkers; (4) elucidation of the relationship between gut microbiota and therapeutic efficacy, including identification of key predictive taxa; (5) standardization of ctDNA, exosome, and other circulating biomarkers for clinical application; (6) integration of multi-omics and AI-based approaches to establish composite predictive systems for dynamic monitoring and individualized scoring; and (7) construction and validation of radiomic prediction models incorporating clinical and molecular data for multidimensional integration. By integrating multidimensional data with AI and ML, a comprehensive predictive framework spanning immune, molecular, metabolic, and imaging dimensions can be established. This approach will enhance the accuracy and stability of immunotherapy (219, 220).

### 5.2 Construction and optimization of predictive models for immunotherapy

With the continuous advancement of biomarker research, the development of scientifically robust, stable, and clinically applicable predictive models has become a pivotal focus in immunotherapy. Researchers have employed multivariate analysis and ML approaches to explore combined biomarker detection strategies, aiming to construct predictive models with higher sensitivity and greater reproducibility to support individualized decision-making in gastric cancer immunotherapy.

Several studies have attempted to combine PD-L1 expression with other biomarkers to construct more precise predictive models of immunotherapy. For example, Chen et al. proposed a combined predictive model integrating PD-L1 expression and characteristics of tumor-infiltrating immune cells, which demonstrated a high accuracy in predicting the response of gastric cancer patients to ICIs (221). In addition, the Immunophenoscore (IPS) developed by The Cancer Immunome Atlas (TCIA) integrates key parameters such as MSI, TMB, and immune cell infiltration to quantitatively assess the immune responsiveness of gastric cancer patients, offering a novel strategy for precisely identifying potential beneficiaries (222). Meanwhile, advances in radiomics have provided an important complementary tool for predicting ICIs efficacy. Recent studies have extracted radiomic features from CT images and combined them with clinical biomarkers to develop models capable of predicting PD-L1 expression levels and tumor immune status (223). In the future, integrating multimodal imaging features with immune-related parameters is expected to facilitate the construction of more broadly applicable predictive models for gastric cancer immune responses (224).

AI, ML and deep learning (DL) have been increasingly applied to the prediction of immunotherapy in oncology. AI-based models can integrate clinical data, biomarkers, genomic information, and radiomic data to uncover latent features that are difficult to identify using traditional statistical methods, thereby improving the accuracy and robustness of efficacy prediction (225). DL shows significant advantages in handling high-dimensional radiomic and genomic data, as it can automatically extract key features and construct efficient nonlinear predictive models. For example, Xu et al. developed a convolutional neural network (CNN)-based model that combined PET/CT features with clinical information, successfully predicting the response of NSCLC patients to PD-1 inhibitors, with an AUC of 0.83, demonstrating strong clinical potential (226). Similarly, Zhang et al. developed a ML model integrating PD-L1, TMB, and peripheral inflammatory markers, which effectively predicted ORR and PFS following ICIs treatment (227). These studies demonstrate that AI holds unique advantages in integrating multimodal data and constructing individualized predictive models (221).

Future predictive models for immunotherapy are expected to evolve along several directions. First, conventional models are mostly based on static pre-treatment information and fail to capture real-time immune dynamics during therapy. Future models will increasingly rely on longitudinal data and dynamic monitoring, utilizing continuously evolving clinical data during treatment to enable early detection and evaluation of therapeutic responses and enhance predictive timeliness and sensitivity (228). Second, integrated analysis of multiple biomarkers will become mainstream. Incorporating multi-omics data, including genomics, transcriptomics, and proteomics, will enhance the biological interpretability and predictive precision of models (17). Moreover, future models must emphasize visualization by presenting feature contributions, risk scores, and individualized response maps to enhance their clinical decision-support value. Finally, future efforts should focus on large-scale, multicenter, and cross-population model training and validation, along with the standardization of data collection and biomarker detection protocols, to enhance model reproducibility and clinical applicability (229). The multimodal predictive models not only provides a theoretical basis for personalized immunotherapy but also underscores the potential of combined biomarker assessment in precision treatment decision-making (230).

### 5.3 Optimization and innovation of combination strategies

Although ICIs have shown promising efficacy in a subset of gastric cancer patients, their overall response rate remains suboptimal. Future research should focus on optimizing combination strategies to synergistically enhance immune activation, and expand the population that benefits from immunotherapy. Currently, the combination of ICIs with chemotherapy, radiotherapy, targeted therapy, and other novel approaches has demonstrated considerable potential, but their underlying mechanisms, efficacy variations, and patient-specific applicability require further investigation.

#### 5.3.1 ICIs combined with chemotherapy

In addition to directly inducing tumor cell apoptosis, chemotherapy can enhance antitumor immune responses through multiple mechanisms. Certain chemotherapeutic agents can induce immunogenic cell death, releasing tumor-associated antigens and “danger signals,” which subsequently promote DC activation and antigen presentation, thereby improving the tumor immune microenvironment (231). Additionally, some chemotherapeutic agents can upregulate PD-L1 expression and create more favorable conditions for ICIs targeting (232). The method of ICIs chemotherapy combination is still in the exploratory phase, and there are still significant differences among patients, with some individuals failing to achieve clear benefit. In addition, the long-term efficacy, optimal patient selection, and underlying mechanisms remain incompletely understood, necessitating further refinement of combination strategies (233).

Future studies should focus on: (1) elucidating the synergistic mechanisms and clarifying the effects of chemotherapy on immune pathways; (2) exploring reasonable medication timing and dosage regimens, and clarifying the impact of “concurrent” or “sequential” drug administration methods on treatment efficacy; and (3) Evaluating the differences in combinations of ICIs with different types of chemotherapeutic drugs, optimizing treatment safety, and seeking more suitable immunotherapy combinations.

#### 5.3.2 ICIs combined with radiotherapy

Radiotherapy not only directly eradicates tumor cells to control local lesions but also induces apoptosis, thereby promoting the release of tumor antigens and the exposure of immunogenic components (234). Moreover, radiotherapy can improve the tumor immune microenvironment by reducing the activity of immunosuppressive cells to enhance the immune system’s ability to recognize and attack tumor cells (235). As a core strategy to relieve immune suppression, ICIs can synergize with radiotherapy at both local and systemic levels, thereby expanding the population of patients who may benefit. However, current clinical practice still lacks a systematic understanding of radiotherapy parameters and their immunomodulatory effects. The optimal dose, fractionation schemes, and irradiation fields for immune activation remain unclear, and the timing of ICIs administration and patient-specific responses require further investigation (236).

Future research should focus on: (1) clarifying the impact of different radiotherapy regimens (such as dosage, fractionation, and irradiation area) on the degree of immune activation, and exploring radiotherapy parameters with more immune synergistic effects; (2) evaluating potential adverse reactions and their management strategies to ensure the safety and feasibility; and (3) assessing the potential of ICIs + radiotherapy in conversion therapy, perioperative period, and high-risk subtypes of recurrence, and expanding indications and applicable populations.

#### 5.3.3 ICIs combined with targeted therapy

The immunosuppressive TME is a major factor limiting the efficacy of ICIs. Aberrant activation of the VEGF signaling pathway not only promotes tumor angiogenesis but also suppresses DC maturation and recruits Tregs and MDSCs, thereby reinforcing the immunosuppressive state (237, 238). Anti-VEGF/VEGFR therapy may “normalize” tumor vasculature, reduce the infiltration of immunosuppressive cells, and enhance effector T-cell infiltration and activity, thereby reshaping the TME to favor immune activation (239). Currently, multiple clinical trials evaluating ICIs-targeted therapy combinations in advanced gastric cancer have demonstrated promising tolerability and clinical benefit. The scope of targeted therapy continues to expand, with an increasing number of novel targets—such as Claudin 18.2, FGFR, and CSF1R—as well as small molecule inhibitors that regulate immune pathways are entering the research of combination therapy (240, 241).

Future research should focus on: (1) screening suitable targets and combination schemes, and clarifying the optimal targeting strategies for different molecular subtypes; (2) exploring reasonable treatment sequences, dosages, and drug combinations to achieve maximum synergistic effects and reduce adverse reactions; and (3) analyzing the molecular mechanisms of ICIs+targeting, exploring the regulatory effects of targeted drugs on TME and immune pathways, and providing theoretical support for target development and combination optimization (242).

#### 5.3.4 Combination of multiple ICIs

The simultaneous blockade of multiple immune checkpoints has emerged as a promising frontier in gastric cancer immunotherapy. Combination therapy with PD-1/PD-L1 and CTLA-4 inhibitors can simultaneously block distinct stages of immunosuppressive signaling, synergistically activating T cells and enhancing tumor recognition and clearance, thereby inducing stronger and more durable antitumor immune responses (243). Clinical studies across various solid tumors have demonstrated superior ORR and enhanced immune memory responses with combination therapy compared to monotherapy, and its therapeutic potential in gastric cancer is increasingly evident. However, excessive immune activation also increases the risk of irAEs, making the balance between efficacy and toxicity a central clinical challenge (244, 245).

Future research should focus on: (1) integrating biomarkers such as TMB, MSI, and PD-L1 to precisely identify populations most likely to benefit; (2) optimizing dosing, initiation timing, and combination strategies—including phased, low-dose, or alternating regimens; and (3) strengthening toxicity monitoring and management to achieve an optimal balance between efficacy and safety.

#### 5.3.5 ICIs combined with other therapies

The combination of ICIs with other novel therapeutic strategies has emerged as a research hotspot in gastric cancer immunotherapy. These approaches aim to enhance antitumor immune responses at multiple levels, and expand the eligible patient population. Combining ICIs with antibody–drug conjugates (ADCs) enables precise targeting of tumor-specific antigens while simultaneously inducing immunogenic cell death, enhancing antigen presentation, and activating adaptive immune responses (246). The combination of ICIs with tumor vaccines or adoptive cell immunotherapy—such as cytokine-induced killer (CIK) cells and chimeric antigen receptor T (CAR-T) cells—can further enhance tumor-specific T-cell responses, fundamentally improving immune recognition and cytotoxicity (247). Moreover, the combination of ICIs with agents targeting the tumor immune microenvironment—such as Treg inhibitors, MDSC inhibitors, or immune metabolism modulators—has the potential to alleviate immunosuppression, and improve T-cell infiltration and function (248). Future research should elucidate the immunological mechanisms and action pathways underlying these novel combination strategies, identify synergistic therapeutic combinations, and promote mechanistic studies and early-phase clinical trials to validate their efficacy and safety.

Combination strategies involving ICIs represent a breakthrough direction for gastric cancer immunotherapy. Future efforts should include the following aspects: (1) conducting clinical studies with large sample sizes and long-term follow-up to verify the stability and safety of therapeutic effects; (2) Screening sensitive biomarkers and identifying patients who may benefit from combination therapy; (3) Exploring the dosage and administration of ICIs in different combination strategies, and developing personalized medication strategies based on the patient’s immune status; (4) Building predictive models to guide individualized application of combination therapy. We should continue to promote the precision and standardization of combination therapy, expand the target population and clinical benefits of immunotherapy.

### 5.4 Targeting resistance mechanisms and immune reprogramming

The resistance to ICIs has become a major challenge limiting their therapeutic efficacy and long-term clinical benefits. To overcome this bottleneck, future research should focus on the precise identification and targeted modulation of resistance mechanisms, remodeling the antitumor immune microenvironment to reactivate immune sensitivity, and developing multidimensional, mechanism-driven strategies for resistance reversal.

To counteract tumor immune evasion, studies have explored approaches to upregulate MHC-I expression and restore antigen presentation, thereby enhancing tumor visibility to T cells. For example, activating the STAT1/IRF1 pathway via RNA interference or small-molecule agonists has been shown to increase MHC-I and TAP1 expression, reversing β2M-loss-induced “immune blindness” and re-initiating T-cell recognition and cytotoxicity (249, 250). Epigenetic modulators, such as DNA methyltransferase inhibitors (DNMTis) and histone deacetylase inhibitors (HDACis), can remodel chromatin accessibility, enhance immune-related gene expression, and improve antigen presentation, thereby augmenting synergy with ICIs (251, 252). Simultaneous blockade of multiple immune checkpoints has also emerged as a promising strategy to address resistance. Several clinical trials are evaluating the safety and efficacy of PD-1 inhibitors combined with LAG-3, TIM-3, or TIGIT antibodies. For instance, relatlimab (anti-LAG-3) plus nivolumab has demonstrated superior response rates compared to monotherapy in melanoma, warranting investigation in gastric cancer (253). Moreover, interventions targeting T-cell exhaustion and metabolic adaptation—such as promoting precursor-exhausted T-cell (Tpex) differentiation into effector T cells and restoring their proliferative and cytokine-secreting capacities—could sustain ICIs-induced immune responses (254).

Remodeling the TME is another key strategy for overcoming resistance. Eliminating immunosuppressive cell populations such as MDSCs and Tregs or inhibiting key suppressive factors like TGF-β and VEGF can significantly enhance CD8^+^ T-cell infiltration and functionality (255, 256). Strategies currently under clinical evaluation include IDO inhibitors (e.g., epacadostat), TGF-β pathway inhibitors (e.g., galunisertib) (257), and VEGF pathway inhibitors (e.g., bevacizumab) (258), which collectively relieve immunosuppression and strengthen T-cell responses. Combining ICIs with such inhibitors to establish a dual “immune activation + immune disinhibition” approach has shown promise in various solid tumors and may develop a mechanistically defined treatment strategy in gastric cancer.

Targeting immune metabolism has also emerged as a cutting-edge strategy. Tumor-derived lactate suppresses T-cell activity by acidifying the TME. The inhibition of lactate dehydrogenase A (LDHA) or monocarboxylate transporter 1 (MCT1) has been shown to restore T-cell cytotoxicity (259). Blocking the CD39/CD73-adenosine-A2A receptor axis may alleviate adenosine-mediated T-cell suppression (260), while IDO and TDO inhibitors targeting tryptophan metabolism have demonstrated potential to enhance immune responses (261). Future work integrating metabolomics may enable the identification of patient-specific metabolic-immune profiles and the development of co-targeting strategies (262). Emerging technologies are providing additional tools to combat resistance. Nanomedicine platforms can deliver small molecules, siRNA, and proteins with high specificity, reducing systemic toxicity while enhancing local drug concentration (263). Synthetic biology approaches can engineer T cells or microbes with microenvironment-responsive activation functions, offering new avenues for localized immune modulation (264).

Future research should advance the following priorities: (1) constructing molecular resistance subtype atlases to define resistance patterns across gastric cancer populations; (2) developing longitudinal monitoring systems that integrate ctDNA, immune cell profiling, and TCR clonality for early detection and dynamic prediction of resistance; (3) conducting large-scale, multicenter clinical validation to identify beneficiary subgroups and translate resistance-reversal strategies into clinical practice; (4) exploring integrated interventions combining immune modulators, metabolic targeting, checkpoint blockade, and engineered delivery systems to achieve systemic immune re-sensitization; and (5) leveraging single-cell sequencing, spatial omics, and longitudinal immunomics to capture immune evolution at high resolution, identify critical resistance transition points, and develop real-time predictive models (265).

### 5.5 Microbiota interventions and personalized microbiome modulation

In recent years, the role of the gut microbiota in regulating the host immune system has gained increasing attention, and microbiota-targeted interventions are emerging as a promising strategy to enhance the efficacy of cancer immunotherapy. Rather than serving merely as predictive biomarkers, active reshaping of the gut microbiome to restore an immune-supportive microbial state offers greater therapeutic potential. Currently, multiple microbiota-modulating approaches — including probiotic supplementation, fecal microbiota transplantation (FMT), dietary modification, synbiotics, antibiotics, and synthetic microbial ecosystems — are under investigation across various cancer types (266). Among these, FMT has emerged as a “fast-track” method for microbiota reconstruction, with breakthrough results observed in melanoma. Baruch et al. demonstrated that transplanting fecal microbiota from PD-1 responders into non-responders significantly enhanced the latter’s response to ICIs, achieving an ORR of 30% (202). Similarly, Davar et al. reported that FMT combined with immunotherapy improved treatment outcomes in melanoma patients (267). These findings offer potentially translatable strategies for gastric cancer. In preclinical gastric cancer models, specific bacterial strains such as Bifidobacterium breve and Lactobacillus rhamnosus GG have been shown to activate the DC–NK–T-cell axis, enhance intratumoral CD8^+^ T-cell infiltration, and improve anti-PD-1 efficacy (199, 268). Additionally, metabolically active probiotics that secrete short-chain fatty acids (e.g., butyrate) or modulate the tryptophan–kynurenine pathway can mitigate immunosuppression within the TME, thereby sustaining ICIs efficacy (269).

However, due to the highly individualized composition and function of the gut microbiota, standardized interventions are insufficient, underscoring the need for precise, personalized modulation strategies. Patient stratification based on microbial composition or functional characteristics — identifying “immune-supportive” and “immune-suppressive” microbiota subtypes — can guide intervention selection (270, 271). At the same time, functional pathway–targeted interventions are being developed, such as dietary supplementation, prebiotic administration, or enzyme inhibition to regulate lactate metabolism, short-chain fatty acid release, or IDO activity (272, 273). Moreover, advances in synthetic biology offer new possibilities for directed microbiota engineering, enabling the creation of multispecies consortia or engineered bacteria for localized immune modulation with enhanced stability and specificity (274).

Despite its promise, microbiota-based interventions face significant clinical challenges: (1) the absence of standardized microbiome analysis and intervention evaluation systems, leading to limited clinical validation; (2) unpredictable post-intervention colonization dynamics and stability, resulting in high variability in therapeutic outcomes; and (3) regulatory, ethical, and biosafety concerns, including donor selection criteria, potential pathogen transmission, and quality control issues. Future research should focus on: (1) establishing standardized microbiome profiling platforms to identify microbial signatures associated with ICIs efficacy; (2) conducting multicenter prospective clinical trials to evaluate efficacy, safety, and synergy with ICIs; (3) developing individualized probiotic formulations or synthetic microbial therapeutics based on “responsive microbiota” selection to achieve controllable, safe, and standardized interventions; and (4) constructing integrated microbiota–immune–ICIs response prediction models to inform patient selection for microbiota-based combination therapies.

Through microbiota-targeted interventions and individualized modulation strategies, it may be possible to overcome the current “non-response bottleneck” in gastric cancer immunotherapy, shifting the paradigm from passive identification of responsive populations to active induction of immune responsiveness. Furthermore, the integration of systems biology, AI, and spatial multi-omics technologies may enable microbiota interventions to synergize with TME remodeling and immunometabolic modulation, paving the way for truly personalized, multi-modal immunotherapeutic strategies.

### 5.6 Prediction and personalized management of immune-related toxicity

With the expanding clinical use of ICIs in gastric cancer, irAEs have emerged as a major factor limiting their long-term application. Because gastric cancer patients often have poor baseline physical condition and multiple comorbidities, irAEs are more likely to involve multiple organs, thereby increasing the risk of treatment discontinuation or even death. The clinical manifestations of irAEs are highly heterogeneous, their underlying mechanisms are complex, and effective predictive approaches remain lacking (275). Future research must urgently focus on developing scientifically robust toxicity risk prediction systems and individualized intervention strategies to achieve a dynamic balance between efficacy and safety.

At the mechanistic level, further elucidation of the immunopathological basis of ICIs-related toxicities is required, including cross-recognition of self-antigens, disruption of self-tolerance, cytokine storms, and tissue-specific T-cell activation. Identifying immune signatures associated with susceptibility is also critical (276). In terms of toxicity prediction, recent studies have attempted to integrate genomic, immunomic, and clinical data to develop irAEs risk assessment models. Certain autoimmune genes, such as specific HLA alleles and polymorphic SNP loci, have been linked to organ-specific toxicities (e.g., immune myocarditis) and may serve as genetic markers of susceptibility (277). Furthermore, peripheral cytokine levels (e.g., IL-6, TNF-α), immune cell subset ratios (e.g., Th17/Treg), TCR clonal expansion trends, and baseline autoantibody profiles are closely associated with irAEs development (278). Future multimodal prediction models integrating these indicators could enable the early identification of high-risk patients and provide reference for dose adjustments or protective combination strategies (279).

Individualized management strategies should focus on targeted interventions tailored to the type and mechanism of toxicity. For example, ICIs-induced colitis can be managed by modulating the gut microbiota or administering IL-1/IL-6 inhibitors; hepatitis-type toxicities may benefit from anti-TNF-α or JAK pathway inhibitors; and endocrine toxicities such as thyroid dysfunction can be treated with hormone replacement therapy while monitoring autoantibody levels to guide decisions about continuing immunotherapy (280). With the emergence of Treg stabilizers, metabolic pathway inhibitors (e.g., IDO1 inhibitors), and cytokine-neutralizing antibodies, future therapies may achieve precise modulation of immune response magnitude and direction, maintaining antitumor efficacy while minimizing toxicity (281). Dynamic monitoring and responsive intervention will also be crucial for personalized toxicity management. Future approaches should leverage wearable devices, AI-assisted platforms, and biomarker-driven early warning systems for early detection and stratified management of irAEs (282). Clinically, collecting longitudinal data on irAEs profiles specific to gastric cancer patients and building dedicated toxicity databases will support toxicity–efficacy co-modeling and enable individualized “risk–benefit” decision tools (283).

Future research should advance in several key directions: (1) systematically characterizing the incidence, temporal evolution, and mechanistic features of irAEs and identifying organ-specific predictive factors; (2) integrating genomic, peripheral immune, and clinical parameters to develop multimodal predictive models; (3) designing mechanism-based interventions that target metabolic regulation, inflammatory pathways, and the immune microenvironment; (4) building intelligent management systems centered on dynamic monitoring, precision warning, and stratified intervention; and (5) exploring mechanistic links between toxicity and efficacy to distinguish “beneficial” from “harmful” toxicities, thereby supporting individualized clinical decision-making. Establishing a mechanism-driven framework for toxicity prediction and intervention could shift gastric cancer immunotherapy from empirical safety monitoring toward precision risk management, ensuring the safe and sustainable use of ICIs. A comprehensive summary of key research priorities, challenges, and future strategies is provided in **Supplementary Table 1**.

**Figure 6:**
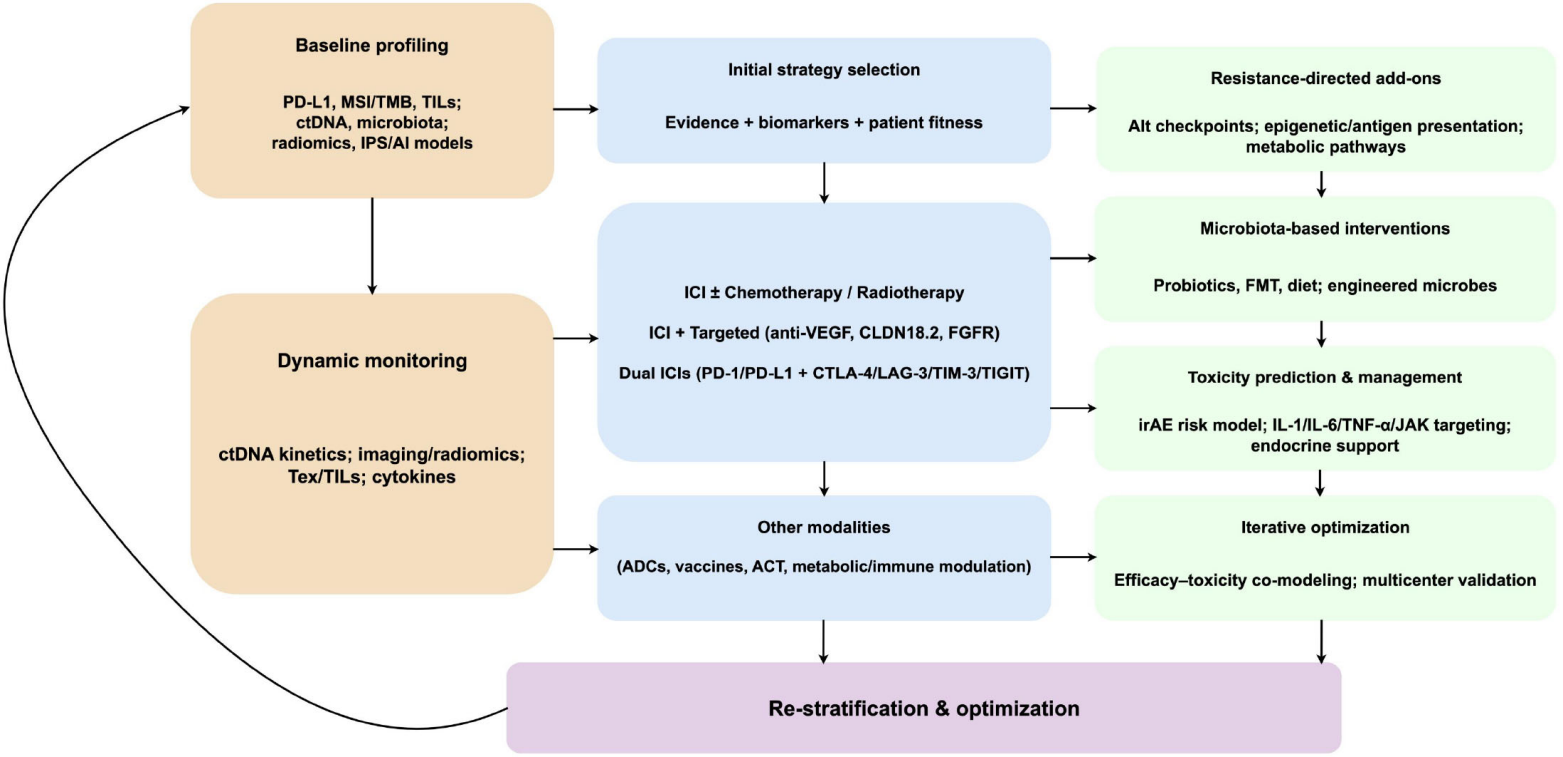
Precision immunotherapy framework and future strategies for optimizing ICI therapy in gastric cancer. This framework integrates baseline profiling (biomarkers, ctDNA, microbiota, radiomics), initial treatment selection (monotherapy, chemotherapy, targeted therapy, or dual ICIs), and dynamic monitoring. Additional layers—resistance-directed interventions, microbiota modulation, toxicity management, and iterative optimization—enable continuous re-stratification and treatment adaptation, aiming to enhance efficacy, overcome resistance, reduce irAEs, and achieve personalized immunotherapy.

As immunotherapy continues to advance in the management of gastric cancer, achieving individualized and precision-based interventions has become a central direction for future research. Previous efforts involving biomarkers, predictive modeling, combination strategies, resistance modulation, microbiota regulation, and immune toxicity management have collectively laid the theoretical and technical foundation for a comprehensive precision immunotherapy framework. Future studies should prioritize the establishment of an integrated framework centered on “precision prediction, dynamic monitoring, and synergistic intervention” to drive gastric cancer immunotherapy toward a more precise, effective, and sustainable paradigm (284, 285) (**Figure 6**).

## 6 Conclusions

ICIs have reshaped the treatment landscape of gastric cancer and have delivered meaningful survival gains for a subset of patients, particularly in biomarker-enriched populations. Nevertheless, durable benefit remains limited by modest activity of monotherapy in unselected cohorts, frequent primary and acquired resistance, the scarcity of robust and standardized predictive biomarkers, the complexity and plasticity of the tumor microenvironment, substantial interpatient heterogeneity, and irAEs. In this narrative review, we summarize current advances in ICIs for gastric cancer across treatment settings, discuss key biological and clinical barriers that constrain efficacy, and highlight emerging strategies aimed at improving patient selection, deepening responses, and maintaining safety.

From a clinical perspective, ICIs are now integral to systemic therapy for advanced/metastatic gastric cancer, largely driven by combination regimens, whereas perioperative applications remain practice-evolving and should be interpreted in the context of maturing phase III evidence. The most consistent benefit is observed in immunologically “inflamed” subgroups—most notably MSI-H/dMMR tumors and, in many settings, patients with higher PD-L1 CPS—supporting biomarker-informed treatment selection. At the same time, the limited efficacy of ICI monotherapy in unselected populations underscores the importance of tumor-intrinsic immune escape, heterogeneous antigenicity, and an immunosuppressive tumor microenvironment. Accordingly, rational combinations (e.g., chemo–IO, anti-angiogenic–IO, IO–targeted, radiotherapy–IO, and selected dual-checkpoint approaches) aim to enhance immunogenicity, improve immune infiltration, and counter adaptive resistance, but require careful attention to toxicity and feasibility. Importantly, single biomarkers are unlikely to be sufficient; clinically useful stratification will probably require integrated frameworks that combine tumor genomics, immune contexture, and longitudinal circulating and imaging readouts.

Looking ahead, several near-term priorities are clinically testable and could accelerate translation, including harmonization and prospective validation of biomarker assays and cutoffs, biomarker-guided trials to optimize combination choices and sequencing with predefined subgroup analyses, standardized longitudinal monitoring (such as ctDNA kinetics) to detect early response and emerging resistance, and risk-stratified prevention and management strategies for irAEs. In parallel, longer-term exploratory directions should focus on single-cell and spatial profiling to map immune–metabolic–stromal resistance networks and identify actionable nodes, microbiota-based predictors and interventions specific to gastric cancer, interpretable multi-omics integration with AI/ML to build generalizable predictive models, and radiomics/imaging biomarkers integrated with molecular and immune features. Collectively, these efforts may sharpen patient selection, rationalize combination strategies, and advance precision immunotherapy for gastric cancer.
