# Supplementary material for: Immune checkpoint inhibitor therapy for gastric cancer: current status, therapeutic challenges, and future prospects
Source: Front Immunol. 2026 Feb 26;17:1716934. doi: 10.3389/fimmu.2026.1716934 (PMC12979166; doi:10.3389/fimmu.2026.1716934)
Supplement: Supplementary file 1 [file DataSheet1.docx]

**Supplementary Table 1 Future research axes, key insights, challenges, and directions in ICIs-based immunotherapy for gastric cancer.**

| Research Axis | Key Insights | Challenges | Future Directions |
| --- | --- | --- | --- |
| Biomarker Research and Application | - Established biomarkers: PD-L1, MSI-H/dMMR, TMB - Emerging biomarkers: TILs (CD8⁺), Tex cells, TME features (MDSCs, Tregs, cytokines, metabolic signatures) - Novel biomarkers: ctDNA, exosomes, gut microbiota, LAG-3/TIM-3/VISTA | - Limited sensitivity and specificity of single biomarkers - Heterogeneity in detection methods and lack of standardized thresholds - Low prevalence of MSI-H/TMB-H (~5–10%) | - Develop integrated biomarker panels and composite scoring systems - Standardize detection platforms and evaluation criteria - multicenter prospective validation - Integrate multi-omics biomarker models |
| Therapeutic Response Prediction Models | - Multimodal integration: PD-L1, MSI/TMB, TILs, IPS - Radiomics: CT/PET features combined with molecular and clinical parameters - AI-based models: ML and deep learning integrating multi-omics data | - Static models fail to capture dynamic immune responses - Limited sample sizes and lack of cross-center validation - Lack of standardized detection and algorithmic pipelines | - Establish dynamic longitudinal predictive models - Standardize data acquisition and detection workflows - Develop AI models and visualization tools - large-scale, multicenter validation |
| Combination Therapy Strategies | - ICIs + Chemotherapy: Induces immunogenic cell death, reduces Tregs/MDSCs - ICIs + Radiotherapy: Promotes antigen release, activates cGAS–STING and type I IFN pathways - ICIs + Targeted therapy: Anti-VEGF, CLDN18.2, FGFR inhibitors remodel the TME - Dual ICIs: PD-1/PD-L1 + CTLA-4/LAG-3/TIM-3/TIGIT blockade - Other approaches: ADCs, cancer vaccines, adoptive cell therapies, metabolic or immune modulation | - Optimal combination regimens, sequencing, and dosing remain undefined - Increased risk of irAEs and treatment-related toxicity - Inadequate patient selection and mechanistic validation - Limited large-scale clinical evidence | - optimization of combination regimens and patient stratification - Exploration of synchronous vs. sequential and dose-adjusted strategies - Enhanced toxicity management and promotion of clinical trials |
| Resistance Mechanisms | - Antigen presentation defects (MHC-I/β2M/TAP loss) and epigenetic modulation (DNMTi, HDACi) - Synergistic checkpoint blockade: PD-1 + LAG-3/TIM-3/TIGIT - Immunosuppressive cells/factors: MDSCs, Tregs, TGF-β, VEGF, IDO - Immunometabolic pathways: lactate, adenosine, tryptophan - Emerging technologies: nanodelivery systems, engineered T cells/microbes | - High heterogeneity of resistance mechanisms and lack of systematic classification - Limited biomarkers for dynamic monitoring - Most strategies remain at preclinical or exploratory stages | - Map molecular subtypes and resistance evolution trajectories - multicenter clinical validation - Explore integrated approaches combining immune, metabolic, and engineering-based interventions |
| Microbiota-Based Interventions | - Approaches: Probiotics, FMT, dietary modulation, synbiotics, engineered microbes - Beneficial taxa: Akkermansia, Bifidobacterium, Faecalibacterium - Functional pathways: SCFAs, tryptophan–kynurenine, lactate metabolism | - High interindividual variability; poor colonization and stability - Lack of standardized detection methods and efficacy evaluation systems - Limited clinical validation; safety and ethical concerns | - Establish microbiota profiling platforms and core feature libraries - multicenter prospective trials - Develop personalized microbial therapeutics and engineered bacteria - Build integrated microbiota–immune–ICI predictive models |
| irAEs Prediction and Toxicity Management | - Mechanisms: Cross-recognition of self-antigens, cytokine storms, tissue-specific T-cell activation - Risk factors: HLA subtypes, SNPs, cytokine signatures, Th17/Treg ratio, TCR expansion, autoantibody profiles - Predictive models: Multi-omics integration, AI-driven risk assessment - Management strategies: Pathway-targeted therapy (IL-1/IL-6, TNF-α, JAK), microbiota modulation, hormone replacement | - Complex mechanisms and heterogeneous clinical manifestations - Lack of standardized prediction tools and risk stratification systems - Difficult to balance efficacy and toxicity | - Develop multimodal risk prediction models and early warning systems - Create stratified intervention and personalized management strategies - Build irAE databases and strengthen toxicity–efficacy co-modeling |
